# Experimental Characterization of Emergent Behavior in Bio-Inspired Swarm Intelligence Algorithms

**DOI:** 10.3390/biomimetics11080576

**Published:** 2026-08-12

**Authors:** Yoslandy Lazo, Broderick Crawford, Gino Astorga, Felipe Cisternas-Caneo, José Barrera-Garcia, Giovanni Giachetti, Ricardo Soto

**Affiliations:** 1 Escuela de Ingeniería Informática, Pontificia Universidad Católica de Valparaíso, Avenida Brasil 2241, Valparaíso 2362807, Chilejose.barrera@pucv.cl (J.B.-G.);; 2 Escuela de Negocios Internacionales, Universidad de Valparaíso, Alcalde Prieto Nieto 452, Viña del Mar 2572048, Chile; 3 Escuela de Negocios y Economía, Pontificia Universidad Católica de Valparaíso, Amunátegui 1838, Viña del Mar 2580129, Chile; 4 Facultad de Ingeniería, Universidad Andres Bello, Antonio Varas 880, Providencia, Santiago 7591538, Chile

**Keywords:** bio-inspired optimization, swarm metaheuristics, emergent behavior

## Abstract

Bio-inspired swarm metaheuristic algorithms constitute a widely used tool for solving complex optimization problems. However, the experimental characterization of their emergent behavior remains a methodological challenge. This study proposes an experimental framework for characterizing emergent behavior through swarm collective dynamics. The framework integrates complementary dynamic indicators and establishes relative diversity loss as a homogeneous criterion for defining equivalent comparison states across different search processes. The framework was evaluated using the Reptile Search Algorithm (RSA) and Draco Lizard Optimizer (DLO) as case studies, with Particle Swarm Optimization (PSO) serving as a reference algorithm. The results showed that swarm collective dynamics were associated with both the mathematical properties of the search landscape and the search mechanisms of each metaheuristic. Furthermore, relative diversity loss enabled the comparison of different metaheuristics within a common reference framework. In RSA, swarm reorganization occurred during the first iterations. DLO exhibited a more gradual evolution, whereas PSO showed an intermediate behavior between both dynamics. The proposed experimental framework provides a methodological basis for the experimental characterization of emergent behavior in swarm metaheuristics.

## 1. Introduction

Bio-inspired metaheuristic algorithms constitute one of the main tools for solving complex optimization problems encountered in science and engineering [1,2]. These methods abstract mechanisms observed in biological systems to construct efficient and scalable search strategies [1,3]. Within this family, swarm-based metaheuristics occupy a prominent position due to their ability to generate emergent collective behaviors through local interactions among agents [4,5]. As a result, these algorithms have been successfully applied to continuous, discrete, and multi-objective optimization problems [4,6]. The study of the mechanisms governing such emergent behaviors is essential for understanding the functioning of new bio-inspired metaheuristics and fostering their development on more rigorous scientific foundations [5,7,8].

The performance of a swarm-based metaheuristic largely depends on the balance between exploration and exploitation [5,9]. Exploration promotes the discovery of new regions of the search space. Exploitation concentrates computational effort around promising solutions [9,10]. For this reason, numerous bio-inspired algorithms incorporate mechanisms intended to regulate the transition between these two behaviors during the optimization process [3,10,11]. Consequently, recent proposals commonly attribute predominant exploration to the early stages and predominant exploitation to the later stages of the search process [9,11,12]. These dynamics constitute emergent behaviors of the complete system rather than inherent properties of individual operators [13,14].

The interpretation of these dynamics is often based on the conceptual design of the search operators defined by the authors [15,16]. Under this approach, certain operators are classified as exploratory and others as exploitative [3,17]. However, the behavior observed during execution emerges from the joint interaction of all algorithmic components and their adaptation to the search landscape [15,17]. Consequently, the existence of operators conceptually associated with exploration or exploitation does not guarantee that these behaviors will effectively emerge during optimization [7,9]. This limitation becomes particularly relevant when dynamic claims are used to justify the design or superiority of new bio-inspired metaheuristics [5,7,9].

Despite this, the experimental validation of claims related to search dynamics remains uncommon in the specialized literature [5,9,10]. Most studies evaluate new bio-inspired metaheuristics using final performance indicators, convergence curves, and statistical tests [3,17]. In contrast, relatively few studies systematically analyze the temporal evolution of population diversity to verify whether the proposed dynamics actually emerge during execution [8,10]. Consequently, there is still no widely adopted methodology for the experimental validation of emergent behaviors in bio-inspired metaheuristics [5,9]. This limitation hinders both the objective evaluation of new algorithms and the scientific interpretation of the mechanisms underlying their behavior. Furthermore, population dynamics are also influenced by the mathematical properties of the search landscape. The influence of these properties on the evolution of diversity and emergent behavior continues to be analyzed primarily from a descriptive perspective.

Population diversity is one of the most widely used indicators for characterizing search dynamics in bio-inspired metaheuristics [13,15]. Its temporal evolution provides evidence of swarm dispersion and is essential for analyzing the exploration–exploitation balance [2,15]. However, this indicator alone cannot distinguish effective exploration from unproductive dispersion [15]. Nor can it differentiate strategic exploitation from premature convergence toward suboptimal regions [13,15]. Consequently, diversity should be interpreted as an observational indicator of the population state [15]. Its analysis requires complementary information to support inferences about the system’s emergent behavior [12,15].

In this context, a methodology is proposed to experimentally validate emergent behaviors in bio-inspired metaheuristics through dynamic indicators of population diversity. As a demonstration of its applicability, the methodology is applied to the analysis of Reptile Search Algorithm (RSA) and Draco Lizard Optimizer (DLO) [18,19]. Both swarm-based metaheuristics describe explicit mechanisms intended to regulate the balance between exploration and exploitation. The analysis is conducted on benchmark functions with distinct structural properties. This design makes it possible to evaluate the consistency of the observed dynamics across different search landscapes. Consequently, RSA and DLO are used as representative case studies to illustrate the scope of the proposed methodology. Its application is not limited to these two metaheuristics.

The objective of this research is to propose and evaluate an experimental framework for characterizing emergent behavior in bio-inspired swarm metaheuristics. To this end, the proposed framework is applied to RSA and DLO as the algorithms under study, while PSO is employed as a widely used reference algorithm in the literature. This evaluation makes it possible to analyze the relationship between the search mechanisms of each metaheuristic and the collective dynamics observed during the optimization process.

The main contributions of this work are as follows:

- A reproducible experimental framework is proposed to characterize emergent behavior in swarm metaheuristics through the analysis of swarm collective dynamics.
- Relative diversity loss is established as a homogeneous criterion for defining equivalent comparison states across metaheuristics, benchmark functions, and dimensions.
- Experimental evidence is provided showing that swarm collective dynamics were associated with both the mathematical properties of the search landscape and the search mechanisms of each metaheuristic.
- It is shown that the integration of complementary dynamic indicators enables the characterization of different properties of collective behavior throughout the search process.

The remainder of this paper is organized as follows. Section 2 reviews the literature on emergent behavior in bio-inspired metaheuristics and the dynamic indicators used to characterize swarm collective dynamics. Section 3 describes the proposed experimental framework, including the evaluated metaheuristics, benchmark functions, dynamic indicators, and experimental setup. Section 4 presents the experimental results obtained using the proposed framework. Section 5 discusses the main experimental findings and analyzes the relationship between the observed collective dynamics, the mathematical properties of the search landscape, and the search mechanisms of the metaheuristics. Finally, Section 6 presents the conclusions and outlines future research directions.

## 2. Related Work

Bio-inspired metaheuristics are commonly designed using metaphors inspired by evolutionary processes and biological and social systems found in nature [3,11]. These metaphors guide the conceptual assignment of roles to search operators [4,20]. Within this framework, components associated with exploration and exploitation are distinguished [17,20]. However, these interpretations are primarily based on algorithm design rather than empirical observation [7,9]. Consequently, the emergent behavior of the system is assumed from its algorithmic structure [5,9].

The evaluation of metaheuristics is mainly based on final performance indicators [21,22]. These include the best objective value obtained, convergence speed, and comparative statistical tests [5,9]. Such metrics make it possible to estimate the overall quality of an algorithm [22]. However, they do not capture the temporal evolution of the search process [8,21]. Consequently, they do not allow the analysis or validation of the emergent behavior of the system during optimization [21,22,23].

Several approaches have been proposed to observe the internal dynamics of metaheuristics during optimization [12,24]. These approaches include analyzing the evolution of individual solutions within the search space and population-dispersion measures [14,24]. Such methods provide indirect evidence of the emergent behavior of the system [24,25]. Nevertheless, their use has been focused primarily on descriptive analyses or adaptive algorithm design [25,26]. Therefore, their application to the formal validation of emergent behavior remains limited [22,27].

Population diversity is widely used as an indicator of the internal structure of a swarm [21,24]. This measure quantifies the dispersion of candidate solutions at each iteration of the search process [21,28]. High diversity values are commonly associated with exploratory algorithmic behavior [21,26]. Low diversity values are interpreted as an intensification of exploitation in promising regions [11,21]. The temporal evolution of diversity has been used as an indirect indicator to infer the emergent behavior of the system [24,26].

Global diversity measures may conceal relevant variations across dimensions of the search space [23,29]. To address this limitation, Cheng et al. proposed dimensional diversity [30]. This approach estimates dispersion independently for each decision variable [23,26]. Subsequently, these estimates are integrated into a representative global indicator [26,30]. This level of disaggregation enables a more precise characterization of the emergent behavior of the system [26,30].

The dimensional diversity proposed by Cheng et al. [30] has been extended to improve its stability and robustness. Hussain et al. [5] replaced the mean with the median as the measure of central tendency. This modification reduces the influence of extreme values and improves the representativeness of the population state [21,31]. The median-based variant has been applied to multiple swarm-based metaheuristics, including PSO, the Firefly Algorithm, and the Whale Optimization Algorithm [9,11]. Reported results show that this metric can identify transitions between search phases [11,23]. It also enables the estimation of relative changes between exploration and exploitation [9,21,23]. Consequently, median-based diversity has been used as a dynamic indicator to characterize population evolution and infer the emergent behavior of the system [24,26].

Several studies have identified population diversity as an indirect indicator of the exploration–exploitation balance [7,15]. This metric quantifies the spatial dispersion of the population but does not characterize the directionality or effectiveness of the search process [7]. Consequently, a reduction in diversity does not distinguish between strategic exploitation and premature convergence [7,32]. Likewise, high population dispersion does not guarantee effective global exploration, as it may also reflect random agent movements [29,32]. The current literature agrees that diversity should be interpreted as an observational indicator of the population state [32]. Therefore, its analysis requires complementary information to characterize the system’s emergent behavior more rigorously [8,15].

The interpretation of emergent behavior also depends on the structural properties of the search landscape [8,33]. Multimodality increases the probability of becoming trapped in local optima [27,34]. Separability conditions the interaction among decision variables [34]. Ruggedness affects the stability of swarm movements [25,33]. Numerical conditioning modifies the sensitivity of the optimization process to perturbations in the search space. These properties have been used to construct evaluation scenarios with progressively increasing difficulty [25]. They have also motivated the development of function sets with complementary characteristics for comparing metaheuristics [35,36]. Recent studies indicate that these properties also influence the temporal evolution of population diversity, suggesting a relationship between landscape structure and the emergent behavior of the system [8,33].

The interpretation of emergent behavior also depends on the structural properties of the search landscape [8,17]. These properties describe the geometry of the optimization space and influence the response of the search operators [33]. Multimodality increases the presence of local attraction regions and raises the probability of convergence toward suboptimal solutions [11,13]. Non-separability introduces dependencies among decision variables and modifies the effectiveness of the search operators [34,36]. Ruggedness increases the local complexity of the landscape and reduces the stability of population trajectories. High conditioning produces different scales of variation among decision variables and increases the difficulty of the search process. Collectively, these properties influence the temporal evolution of population diversity and the manifestation of emergent behavior [8,33]. However, the relationship among landscape structure, agent dynamics, and the evolution of diversity still lacks a systematic characterization [8,33].

In this context, dynamic diversity indicators have been used primarily to describe the behavior of individual metaheuristics [5,9,23]. They have also been employed to design adaptive control mechanisms [28,31]. However, these approaches were not conceived to validate hypotheses regarding emergent behavior. Nor is there a standard methodological protocol for contrasting observed dynamics with conceptual design claims. This limitation restricts the scientific interpretation of the emergent behavior of the system [5,7].

To overcome this limitation, a methodology is required that quantifies dynamics associated with exploration and exploitation in a verifiable manner. Furthermore, it must enable the comparison of design hypotheses with empirical observations obtained during optimization. In this context, the present work proposes a methodology based on dynamic indicators of population diversity.

## 3. Materials and Methods

### 3.1. General Structure of Metaheuristics

Swarm-based metaheuristics are iterative processes that enable the exploration of a continuous search space through a population of agents. Each agent represents a candidate solution by means of a decision vector:

(1) $$x=\left(x_{1},\ldots ,x_{d}\right)\in R^{d},$$

where *d* denotes the dimensionality of the problem. Agents are interpreted as points in the search space, whose positions are updated at each iteration through interaction and individual evaluation mechanisms.

The swarm dynamics combine global exploration of the feasible search space with local exploitation of promising regions, progressively concentrating the search in high-quality regions.

In this context, the optimization problem is formulated as:

(2) $$\begin{array}{c} \min f(x)\\ \mathrm{subject}\;\mathrm{to}\;x\in S \end{array}$$

where $f:R^{d}\to R$ is the objective function, which assigns a quality value to each agent and guides the swarm dynamics. In this study, the evaluation instances are defined through benchmark functions. The feasible set $S\subseteq R^{d}$ defines the search space:

(3) $$S=\left\{x\in R^{d}|lb_{i}\leq x_{i}\leq ub_{i},\;i=1,\ldots ,d\right\},$$

where $lb_{i}$ and $ub_{i}$ are the lower and upper bounds of dimension *i*, respectively. This set defines the feasible domain within which agents can explore and optimize the objective function. Within the space S, a population of *n* agents is considered, represented by:

(4) $$X^{\left(t\right)}=\left[\begin{array}{c} x_{1}^{\left(t\right)}\\ x_{2}^{\left(t\right)}\\ \vdots \\ x_{n}^{\left(t\right)} \end{array}\right]\in R^{n\times d},\;x_{k}^{\left(t\right)}\in S,\;k=1,\ldots ,n$$

The population $X^{\left(t\right)}$ describes the state of the swarm at iteration *t*. Each $x_{k}^{\left(t\right)}$ represents an agent (candidate solution) whose evaluation contributes to the swarm dynamics. Based on this representation, the population is iteratively updated through the generation of a new population:

(5) $$X^{\left(t+1\right)}=\left[\begin{array}{c} x_{1}^{\left(t+1\right)}\\ x_{2}^{\left(t+1\right)}\\ \vdots \\ x_{n}^{\left(t+1\right)} \end{array}\right]\in R^{n\times d}$$

where each $x_{k}^{\left(t+1\right)}\in S$ corresponds to the updated position of an agent. These positions are generated through one or more movement operators, defined by update rules that make use of the information available in $X^{\left(t\right)}$ and the evaluations of the objective function *f*. Such operators may act individually or in combination, inducing a sequence $X^{\left(t\right)}t\geq 0$ that describes the iterative behavior of the swarm, combining exploration of the search space with exploitation of promising regions. In operational terms, given a stopping criterion, the process is executed for a finite number of iterations (T∈N), generating the finite sequence ${X^{\left(t\right)}}_{t=0}^{T}$. In compact form, the swarm dynamics can be represented by a set of movement operators:

(6) $$O=\{O_{1},\ldots ,O_{m}\},$$

which encapsulate the different interaction, exploration, and exploitation mechanisms that govern the dynamics of the agents. In practice, these operators are combined to define update rules that determine the transition of the population between iterations.

The behavior of the iterative process is determined by a set of control parameters, which influence the generation of the initial population, the dynamics governing the application of the operators, and the stopping criteria. These parameters are represented by a vector:

(7) $$\theta =\left(\theta_{1},\ldots ,\theta_{p}\right)\in \Theta \subseteq R^{p},$$

where *p* denotes the number of algorithm parameters and Θ the space of admissible configurations. Within this framework, the dynamics of the process are determined by the set of movement operators and the algorithm parameters, which govern the swarm’s exploration and exploitation behavior.

Taken together, the initial population $X^{\left(0\right)}$, the movement operators O, the objective function *f*, the parameters θ, and the number of iterations *T* completely define the swarm-based optimization model. This model naturally induces an iterative numerical solution procedure based on update rules applied to the population of agents.

The iterative procedure begins with the initialization of the algorithm parameters θ∈Θ and the generation of the agent population $X^{\left(0\right)}=\{x_{k}^{\left(0\right)}{\}}_{k=1}^{n}\subseteq S$, where each $x_{k}^{\left(0\right)}\in S$ represents an initial agent. At each iteration *t*, the population is evaluated using the objective function, yielding:

(8) $$f^{\left(t\right)}=f\left(X^{\left(t\right)}\right),$$

where $f(X^{\left(t\right)})$ denotes the evaluation of the objective function applied to each agent in the population:

$$f^{\left(t\right)}=\left[\begin{array}{c} f(x_{1}^{\left(t\right)})\\ \vdots \\ f(x_{n}^{\left(t\right)}) \end{array}\right]\in R^{n}.$$

This information is used to guide the population update through the application of movement operators, thereby determining the swarm dynamics. In general, each metaheuristic employs a subset of operators $O^{\prime }\subseteq O$, whose cardinality may vary depending on the algorithm under consideration, with $1\leq \;|O^{\prime }|\;\leq m$. Applied to $X^{\left(t\right)}$, these operators collectively define the update rules that generate the new population $X^{\left(t+1\right)}$.

The process is repeated until a stopping criterion is satisfied, defined by a maximum number of iterations *T*, convergence conditions, or problem-specific constraints. Upon completion of this process, the best solution found during the search is identified:

(9) $$x^{*}=\arg\min_{\begin{array}{c} 0\leq t\leq T\\ 1\leq k\leq n \end{array}}f\left(x_{k}^{\left(t\right)}\right)$$

**Figure 1:**
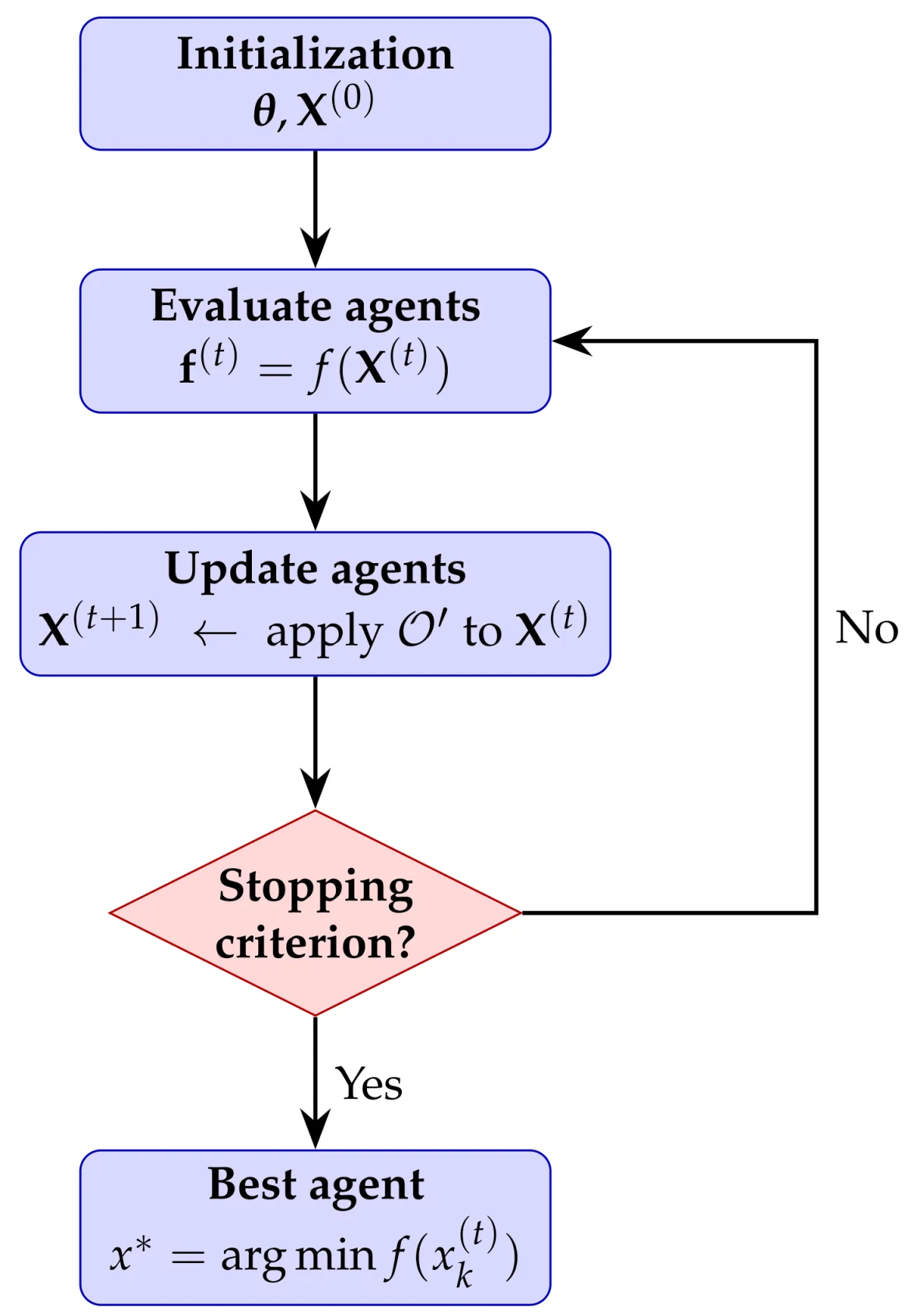
Compact iterative scheme of swarm intelligence-based metaheuristics.

The general structure of the iterative procedure described above can be represented schematically by the flowchart shown in Figure 1, which summarizes the main stages of the swarm-based optimization process.

### 3.2. Particle Swarm Optimization (PSO)

The Particle Swarm Optimization (PSO) algorithm, proposed by Kennedy and Eberhart [37], is a swarm-based metaheuristic inspired by the collective behavior of bird flocks and fish schools during food foraging. The search process is based on cooperation among individuals and the indirect exchange of information regarding the best solutions found.

Each particle represents a candidate solution defined by its position $x_{i}^{\left(t\right)}\in S$ and its velocity $v_{i}^{\left(t\right)}\in R^{d}$. The population is randomly initialized within the continuous search space and is represented by $X^{\left(t\right)}\in R^{n\times d}$. The quality of each solution is determined by evaluating the objective function $f(x_{i}^{\left(t\right)})$. Each particle stores the best personal position attained throughout its trajectory, denoted by ${\mathrm{pbest}}_{i}$, while the swarm maintains the best global position, gbest.

The interaction among particles is achieved through the exchange of global information and individual memory. Each agent updates its trajectory by simultaneously considering its own best experience and the best solution known to the swarm. This mechanism combines individual learning and collective cooperation throughout the iterative search process.

The implementation employed in this study uses the constriction factor model proposed by Clerc and Kennedy [38]. Let $c_{1}$ and $c_{2}$ denote the cognitive and social coefficients, respectively. Based on these parameters, the following quantity is defined

$$\phi =c_{1}+c_{2},$$

and the constriction factor is defined as

(10) $$\chi =\frac{2}{\left|2-\phi -\sqrt{\phi^{2}-4\phi }\right|}.$$

The velocity of each particle is updated according to

(11) $$v_{i}^{\left(t+1\right)}=\chi \left(v_{i}^{\left(t\right)}+c_{1}r_{1}⊙\left({\mathrm{pbest}}_{i}-x_{i}^{\left(t\right)}\right)+c_{2}r_{2}⊙\left(\mathrm{gbest}-x_{i}^{\left(t\right)}\right)\right)$$

where $r_{1}$ and $r_{2}$ are vectors whose components are independent random variables uniformly distributed over [0,1], and ⊙ denotes the element-wise product. The first term preserves the inertial component associated with the previous movement. The second term directs the search toward the particle’s best personal position. The third term guides the movement toward the swarm’s best global solution.

To control the magnitude of the displacement, the velocity of each particle is bounded by a maximum value defined as:

(12) $$v_{\max}=\alpha \left(u-l\right)$$

where u and l denote the upper and lower bounds of the search space, respectively. The coefficient α determines the maximum allowable movement amplitude and remains constant throughout each run. The updated velocity is limited component-wise to the interval $\left[-v_{\max},v_{\max}\right]$ to prevent excessively large displacements during the search.

The position of each particle is updated according to

(13) $$x_{i}^{\left(t+1\right)}=x_{i}^{\left(t\right)}+v_{i}^{\left(t+1\right)}.$$

The new position of each particle is projected onto the feasible search space whenever any of its components exceeds the prescribed bounds. This operation ensures that all solutions remain within the defined search domain. After evaluating the objective function, the personal best position ${\mathrm{pbest}}_{i}$ is updated whenever the new position improves the objective function value. Similarly, the global best position gbest is updated whenever a particle finds a solution with a better objective value than the one currently stored by the swarm.

The overall implementation and execution flow of the PSO algorithm used in this work are summarized in the pseudocode presented in Algorithm 1.
**Algorithm 1** Particle Swarm Optimization (PSO)  1:Initialize population $X^{\left(0\right)}$ within the search space.  2:Initialize particle velocities $V^{\left(0\right)}$.  3:Evaluate $f(x_{i}^{\left(0\right)})$ for all particles.  4:Set ${\mathrm{pbest}}_{i}=x_{i}^{\left(0\right)}$.  5:Set gbest as the best ${\mathrm{pbest}}_{i}$.  6:Compute $v_{max}$ according to Equation (12).  7:**while** t<T **do**  8:      **for** i=1 to *N* **do**  9:        Generate random vectors $r_{1}$ and $r_{2}$.10:        Update velocity according to Equation (11).11:        Limit velocity components within $\left[-v_{max},v_{max}\right]$.12:        Update position according to Equation (13).13:        Project position into the feasible search space.14:        Evaluate $f(x_{i}^{\left(t+1\right)})$.15:        **if** $f(x_{i}^{\left(t+1\right)})$ improves $f({\mathrm{pbest}}_{i})$ **then**16:           Update ${\mathrm{pbest}}_{i}$.17:        **end if**18:        **if** $f({\mathrm{pbest}}_{i})$ improves f(gbest) **then**19:           Update gbest.20:        **end if**21:    **end for**22:    t=t+1.23:**end while**24:**return** gbest.

### 3.3. Reptile Search Algorithm (RSA)

The Reptile Search Algorithm (RSA) was developed based on the hunting and locomotion behavior of reptiles, particularly crocodiles [18]. This behavior was abstracted as a sequential process of exploration and refinement within a continuous search space. The natural dynamics were modeled through movement phases dependent on the iterative progress of the algorithm, with the aim of balancing global exploration and local exploitation during the search for solutions.

Each agent $x_{\left(i,j\right)}^{\left(t\right)}\in S$ is interpreted as a candidate solution to the optimization problem. The population $X^{\left(t\right)}\in R^{n\times d}$ represents the collective state of the search process at iteration *t*. The quality of each agent is evaluated through the objective function $f(x_{\left(i,j\right)}^{\left(t\right)})$. The swarm dynamics are governed by the best solution identified up to the current iteration, denoted by the authors as $Best_{j}\left(t\right)$. Agent interaction is indirect and mediated by this global reference.

The initialization of the algorithm is based on the prior definition of the control parameters α and β. These parameters, fixed at α=0.1 and β=0.1 based on the authors’ sensitivity analysis, are set before the iterative process begins and remain constant throughout the execution. Their configuration determines the exploration intensity of the swarm within the search space. The initial population $X^{\left(0\right)}\subseteq S$ is generated randomly within the feasible domain.

The update of the agents is defined through movement operators that depend on the current iteration *t* and the maximum number of iterations *T*. The process is structured into four stages delimited by the temporal thresholds T/4, T/2, and 3T/4, which are grouped into two main phases: exploration and exploitation. In the following equations, $x_{\left(i,j\right)}^{\left(t\right)}$ represents the *j*-th component of agent *i* at iteration *t*.

During the exploration phase (t⩽T/2), the positions of the agents are updated according to:

(14) $$x_{\left(i,j\right)}^{\left(t+1\right)}=\left\{\begin{array}{cc} Best_{j}^{\left(t\right)}\times -\eta_{\left(i,j\right)}^{\left(t\right)}\times \beta -R_{\left(i,j\right)}^{\left(t\right)}\times rand, & t⩽\frac{T}{4}\\ Best_{j}^{\left(t\right)}\times x_{\left(r_{1},j\right)}\times ES^{\left(t\right)}\times rand, & \frac{T}{4}<t⩽\frac{T}{2} \end{array}\right.$$

During the exploitation phase (t>T/2), the update dynamics are directed toward local refinement around the best global solution, denoted as ${\mathrm{Best}}_{j}^{\left(t\right)}$. In this case, the following update rule is employed:

(15) $$x_{\left(i,j\right)}^{\left(t+1\right)}=\left\{\begin{array}{cc} Best_{j}^{\left(t\right)}\times P_{\left(i,j\right)}^{\left(t\right)}\times rand, & \frac{T}{2}<t⩽\frac{3T}{4}\\ Best_{j}^{\left(t\right)}-\eta_{\left(i,j\right)}^{\left(t\right)}\times \epsilon -R_{\left(i,j\right)}^{\left(t\right)}\times rand, & \frac{3T}{4}<t⩽T \end{array}\right.$$

The operators $\eta_{\left(i,j\right)}^{\left(t\right)}$, $R_{\left(i,j\right)}^{\left(t\right)}$, $P_{\left(i,j\right)}^{\left(t\right)}$, and $ES^{\left(t\right)}$ correspond to the mechanisms defined in the original RSA formulation. In particular, $\eta_{\left(i,j\right)}^{\left(t\right)}$ represents the hunting operator, $R_{\left(i,j\right)}^{\left(t\right)}$ controls the progressive reduction in the search space, $P_{\left(i,j\right)}^{\left(t\right)}$ quantifies the relative difference with respect to the best global solution, and $ES^{\left(t\right)}$ models the evolutionary state of the algorithm. These operators are computed according to the expressions proposed by Abualigah et al. [18], while rand∈[0,1] introduces stochasticity into the search process.

Since the original formulation of RSA, it has been assumed that the algorithm’s behavior changes throughout the iterative process. During the early stages, the combination of rand, $R_{\left(i,j\right)}^{\left(t\right)}$, and $ES^{\left(t\right)}$ favors large displacements within the search space, promoting exploratory behavior. In contrast, during later stages, the dominant influence of $Best_{j}^{\left(t\right)}$, together with the operators $P_{\left(i,j\right)}^{\left(t\right)}$ and $\eta_{\left(i,j\right)}^{\left(t\right)}$, induces a more intensive exploitation behavior around promising regions.

Consequently, the balance between exploration and exploitation is established implicitly through the current iteration *t* relative to the maximum number of iterations *T*. The transition between these two behaviors does not rely on explicit adaptive mechanisms, but rather on predefined temporal intervals that determine the update strategy employed by the agents. This structure constitutes the theoretical foundation that will be experimentally evaluated in this work to verify the actual consistency of these phases.

The general implementation and detailed workflow of RSA, incorporating the exploration and exploitation mechanisms described above, are summarized in the pseudocode presented as Algorithm 2.
**Algorithm 2** Reptile Search Algorithm (RSA)  1:Initialize RSA parameters α,β and initialize the population.  2:**while** t<T **do**  3:      Calculate the fitness of individuals and the global best individual.  4:      Update the ES     **The beginning of the RSA**  5:      **for** i=1 **to** *N* **do**  6:            **for** j=1 **to** *D* **do**  7:               Update the $\eta_{\left(i,j\right)}^{\left(t\right)}$, $R_{\left(i,j\right)}^{\left(t\right)}$, $P_{\left(i,j\right)}^{\left(t\right)}$.                       **Exploration phase**  8:               **if** $\left(t⩽\frac{T}{4}\right)$ **then**  9:                   $x_{\left(i,j\right)}^{\left(t+1\right)}=Best_{j}^{\left(t\right)}\times -\eta_{\left(i,j\right)}^{\left(t\right)}\times \beta -R_{\left(i,j\right)}^{\left(t\right)}\times rand$                        *High walking*10:               **else if** $\left(t⩽2\frac{T}{4}\right)$ **and** $\left(t>\frac{T}{4}\right)$ **then**11:                   $x_{\left(i,j\right)}^{\left(t+1\right)}=Best_{j}^{\left(t\right)}\times x_{\left(r_{1},j\right)}\times ES^{\left(t\right)}\times rand$                                *Belly walking*                       **Exploitation phase**12:               **else if** $\left(t⩽3\frac{T}{4}\right)$ **and** $\left(t>2\frac{T}{4}\right)$ **then**13:                   $x_{\left(i,j\right)}^{\left(t+1\right)}=Best_{j}^{\left(t\right)}\times P_{\left(i,j\right)}^{\left(t\right)}\times rand$                              *Hunting Coordination*14:               **else**15:                   $x_{\left(i,j\right)}^{\left(t+1\right)}=Best_{j}^{\left(t\right)}-\eta_{\left(i,j\right)}^{\left(t\right)}\times \epsilon -R_{\left(i,j\right)}^{\left(t\right)}\times rand$               *Hunting cooperation*16:               **end if**17:            **end for**18:      **end for**19:      t=t+120:**end while**21:**return** the global best.

### 3.4. Draco Lizard Optimizer (DLO)

The Draco Lizard Optimizer (DLO), proposed by Wang, X. [19], is a swarm-based metaheuristic inspired by the behavior of the draco lizard. The algorithm primarily models two biological behaviors observed in this organism. Gliding flight is associated with evasive displacement mechanisms in response to predators, whereas camouflage is linked to strategies of persistence and adaptation within safe regions of the environment. Both behaviors are abstracted as complementary processes of global exploration and local exploitation. This functional duality forms the basis of the two-stage search strategy and the formulation of the DLO algorithm.

The agents $x_{i}^{\left(t\right)}\in S$ are randomly initialized within the continuous search space. Each agent is modeled as a candidate solution. The set of agents at iteration *t* constitutes the population $X^{\left(t\right)}\in R^{n\times d}$, which describes the dynamic state of the swarm throughout the optimization process. The quality of each solution is determined by evaluating the objective function $f(x_{i}^{\left(t\right)})$, which assigns a scalar performance value to each agent. Throughout the iterative process, the best solution found is retained and used as the global reference of the swarm. This solution guides the update of the agents and directs the progressive concentration of the search toward higher-quality regions within the feasible space.

The search strategy of DLO is structured into two distinct temporal phases, which represent the gliding flight and camouflage behaviors of the draco lizard. During the first half of the iterations, global exploration of the search space is emphasized. At this stage, the agents simulate the gliding flight used by the lizard to evade predators. To introduce dynamism into the movement direction and promote trajectory diversification, a directional vector ($\vec{DL}$) is defined:

(16) $$\vec{DL}=\left[DL^{1},DL^{2},\ldots ,DL^{D}\right]$$

whose components are determined by:

$$DL^{j}=\left\{\begin{array}{cc} 1, & \mathrm{if}\;t<\varphi_{1}\;\cdot \;T_{max}\\ P1(k), & \mathrm{else}\;t<\varphi_{2}\;\cdot \;T_{max}\\ P2(k), & \mathrm{else}\;t<\varphi_{3}\;\cdot \;T_{max}\\ P3(k), & \mathrm{else}\;t<\varphi_{4}\;\cdot \;T_{max}\\ 0, & \mathrm{else}\;t<\varphi_{5}\;\cdot \;T_{max} \end{array}\right.\;\forall j=1,2,\ldots ,D$$

where ($P_{1}$), ($P_{2}$), and ($P_{3}$) correspond to binary vectors, (*k*) is a random integer in the interval ([1, 4]), $T_{max}$ represents the maximum number of iterations, and ($\varphi_{1}=0.1$), ($\varphi_{2}=0.2$), ($\varphi_{3}=0.3$), ($\varphi_{4}=0.4$), and ($\varphi_{5}=0.5$). This mechanism progressively modifies the active components of the agents’ displacement, introducing directional variability and promoting the exploration of distant regions within the feasible space.

The position update of each agent during this exploration phase ($t<T_{max}/2$) is performed according to the following rule:

(17) $${\vec{x}}_{i}^{\left(t+1\right)}=\left\{\begin{array}{cc} {\vec{xbest}}_{i}+r\;\cdot \;\left({\vec{x}}_{m}^{\left(t\right)}-A\;\cdot \;{\vec{xbest}}_{i}\right)+\vec{DL}\;\cdot \;r\;\cdot \;\left({\vec{x}}_{m}^{\left(t\right)}-{\vec{x}}_{n}^{\left(t\right)}\right), & \mathrm{if}\;{\vec{xbest}}_{i}>{\vec{xbest}}_{m}\\ {\vec{xbest}}_{i}+r\;\cdot \;\left({\vec{xbest}}_{i}-{\vec{x}}_{m}^{\left(t\right)}\right)+\vec{DL}\;\cdot \;r\;\cdot \;\left({\vec{x}}_{m}^{\left(t\right)}-{\vec{x}}_{n}^{\left(t\right)}\right), & \mathrm{otherwise} \end{array}\right.$$

where $\vec{xbest}i$ is the historical best position of agent *i*, ${\vec{x}}_{m}^{\left(t\right)}$ and ${\vec{x}}_{n}^{\left(t\right)}$ are the positions of two randomly selected agents, *A* is a random parameter that takes the values 1 or 2, *r* is a uniformly distributed random number in [0,1], and $\vec{DL}$ is the dynamic direction vector defined previously.

During the second half of the iterations, local exploitation of the most promising regions identified previously is prioritized. This phase models the camouflage behavior and evasive maneuvers exhibited by the draco lizard when it remains within safe areas of its environment.

The search is concentrated around the best global solution found through adaptive mechanisms aimed at the progressive refinement of candidate solutions. To reduce the risk of stagnation during local exploitation, a Lévy flight operator is incorporated, defined as:

(18) $$levy=0.05\;\cdot \;\frac{u\;\cdot \;\rho }{{\left|\nu \right|}^{\frac{1}{\beta }}},\;\rho ={\left(\frac{\Gamma \left(1+\beta \right)\;\cdot \;\sin\left(\frac{\pi \;\cdot \;\beta }{2}\right)}{\Gamma \left(\frac{1+\beta }{2}\right)\;\cdot \;\beta \;\cdot \;2^{\frac{\beta -1}{2}}}\right)}^{\frac{1}{\beta }}$$

where β=1.5, u=0.5, and $\Gamma \left(x\right)=\left(x-1\right)!$. The position update of each agent during this exploitation phase ($t⩾T_{max}/2$) is performed according to the following rule:

(19) $${\vec{x}}_{i}^{\left(t+1\right)}=\left\{\begin{array}{cc} \vec{gbest}+levy\left(D\right)\;\cdot \;\left(\vec{gbest}-{\vec{x}}_{l}^{\left(t\right)}\right), & \mathrm{if}\;rand(0,1)<p\\ \vec{gbest}+g\;\cdot \;\left(1-\frac{t}{T_{\max}}\right)\;\cdot \;\left({\vec{xbest}}_{i}-{\vec{x}}_{l}^{\left(t\right)}\right), & \mathrm{otherwise} \end{array}\right.$$

where $\vec{gbest}$ represents the best global solution found, ${\vec{xbest}}_{i}$ is the historical best solution of agent *i*, ${\vec{x}}_{l}^{\left(t\right)}$ is the current position of the agent, levy(D) is a vector generated by the Lévy function, *g* is a random number following a normal distribution (mean 0, variance 10), and p=0.2 is the probability of applying a Lévy jump.

This formulation enables the combination of large-amplitude jumps with smaller-scale directed movements, reinforcing intensive exploitation around the best global solution while maintaining the ability to escape from premature stagnation.

After applying the position update rule at each iteration, a greedy selection strategy is employed. This strategy updates the personal best position of each agent, ${\vec{xbest}}_{i}$, if the new solution satisfies $f\left({\vec{x}}_{i}^{\left(t+1\right)}\right)<f\left({\vec{xbest}}_{i}\right)$. Similarly, the global best solution, $\vec{gbest}$, is updated whenever $f\left({\vec{x}}_{i}^{\left(t+1\right)}\right)<f\left(\vec{gbest}\right)$ for some agent *i*. Otherwise, the previous positions are retained.

(20) $${\vec{xbest}}_{i}=\left\{\begin{array}{cc} {\vec{x}}_{i}^{\left(t+1\right)}, & \;\mathrm{if}\;{\vec{x}}_{i}^{\left(t+1\right)}<f\left({\vec{xbest}}_{i}\right)\\ {\vec{xbest}}_{i}, & \;\mathrm{otherwise} \end{array}\right.$$

(21) $$\vec{gbest}=\left\{\begin{array}{cc} {\vec{x}}_{i}^{\left(t+1\right)}, & \;\mathrm{if}\;{\vec{x}}_{i}^{\left(t+1\right)}<f\left(\vec{gbest}\right)\\ \vec{gbest}, & \;\mathrm{otherwise} \end{array}\right.$$

This greedy mechanism guarantees the strict preservation of the best solutions found, promoting the convergence of the swarm toward higher-quality regions.

The general implementation and execution flow of DLO are summarized in the pseudocode presented in Algorithm 3.
**Algorithm 3** Pseudocode of the DLO algorithm  1:Initialize DLO parameters and initialize the population.  2:Calculate the fitness value of the agents  3:Update global optimal fitness value $f_{gbest}$ and the optimal individual position $\vec{xbest}$  4:**while** $t<T_{max}$ **do**  5:      **for** i←1 **to** *N* **do**                  **Exploration phase**  6:            **if** $\left(t<\frac{T_{max}}{2}\right)$ **then**  7:                 $A=\mathrm{round}\left(1+\mathrm{rand}(0,1)\right)$  8:                 initialize the direction vector $\vec{DL}$  9:                 **for** j←1 **to** *D* **do**10:                     update $DL^{\left(j\right)}$11:                 **end for**12:                 **if** ${\vec{xbest}}_{i}>{\vec{xbest}}_{m}$ **then**13:                     ${\vec{x}}_{i}^{\left(t+1\right)}={\vec{xbest}}_{i}+r\;\cdot \;\left({\vec{x}}_{m}^{\left(t\right)}-A\;\cdot \;{\vec{xbest}}_{i}\right)+\vec{DL}\;\cdot \;r\;\cdot \;\left({\vec{x}}_{m}^{\left(t\right)}-{\vec{x}}_{n}^{\left(t\right)}\right)$14:                 **else**15:                     ${\vec{x}}_{i}^{\left(t+1\right)}={\vec{xbest}}_{i}+r\;\cdot \;\left({\vec{xbest}}_{i}-{\vec{x}}_{m}^{\left(t\right)}\right)+\vec{DL}\;\cdot \;r\;\cdot \;\left({\vec{x}}_{m}^{\left(t\right)}-{\vec{x}}_{n}^{\left(t\right)}\right)$16:                 **end if**                  **Exploitation phase**17:            **else**18:                 update *Lévy flight search strategy*19:                 **if** rand(0,1)<p **then**20:                     ${\vec{x}}_{i}^{\left(t+1\right)}=\vec{gbest}+levy\left(D\right)\;\cdot \;\left(\vec{gbest}-{\vec{x}}_{l}^{\left(t\right)}\right)$21:                 **else**22:                     ${\vec{x}}_{i}^{\left(t+1\right)}=\vec{gbest}+g\;\cdot \;\left(1-\frac{t}{T_{\max}}\right)\;\cdot \;\left({\vec{xbest}}_{i}-{\vec{x}}_{l}^{\left(t\right)}\right)$23:                 **end if**24:          **end if**25:          update $\vec{xbest_{i}}$ using greedy selection strategy26:          update $\vec{gbest}$ using greedy selection strategy27:      **end for**28:      t=t+129:**end while**30:**Output:** the global optimal fitness value $f_{gbest}$ and the global optimal position $\vec{gbest}$

### 3.5. Benchmark Functions for Exploration-Exploitation Evaluation

To analyze the dynamic behavior of the metaheuristics under different optimization scenarios, a benchmark set was selected. It consists of functions with complementary mathematical properties. The Sphere, Rosenbrock, Rastrigin, Ackley, and Griewank functions were selected due to the structural diversity of their search landscapes. The selection was motivated by the need to induce different exploration and exploitation demands during the optimization process. Consequently, the benchmark set provides an experimental framework for evaluating phase transitions in RSA and DLO.

The characterization of the benchmark set was performed by considering modality, separability, ruggedness, and conditioning as properties of the search landscape. Problem dimensionality was included as an experimental design criterion to evaluate the scalability of algorithmic behavior. These properties determine the difficulty of the search process and the resulting exploration-exploitation dynamics. Modality determines the distribution of optima within the search space. Separability conditions the interaction among variables. Ruggedness and conditioning influence convergence stability.

**Figure 2:**
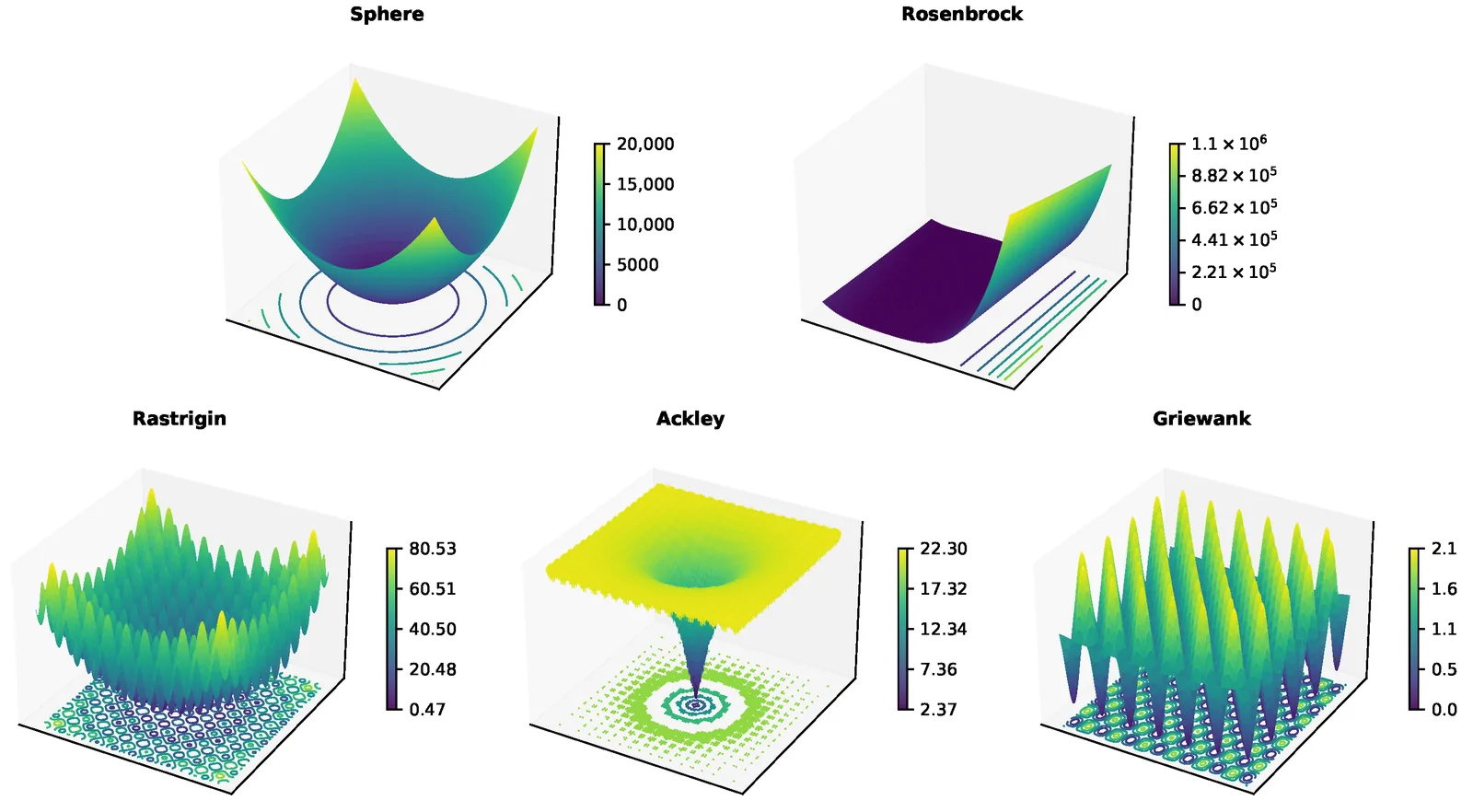
Three-Dimensional Visualization of the Selected Benchmark Functions.

The Sphere function represents a unimodal and separable scenario that serves as a reference for analyzing convergence in simple landscapes. Rosenbrock was incorporated because of its non-separable structure and pronounced conditioning. Its landscape presents a narrow valley that hinders efficient exploitation. Rastrigin provides a highly multimodal and rugged landscape with numerous regularly distributed local optima. Ackley introduces a multimodal structure with high deceptiveness for algorithms guided by local information. Griewank exhibits multimodality combined with moderate interactions among variables. The structural differences among the considered landscapes are illustrated in Figure 2 through their three-dimensional representations.

The selected benchmark set provides broad coverage of continuous optimization scenarios. It combines unimodal and multimodal landscapes, separable and non-separable functions, as well as different levels of ruggedness and conditioning. This diversity reduces biases associated with particular search-space structures and promotes a more robust evaluation of algorithmic behavior. Furthermore, the complementarity among the functions makes it possible to analyze the response of RSA and DLO under substantially different search demands.

The properties represented by this benchmark set generate clearly differentiated search conditions. Quantifying the algorithmic response under these conditions requires indicators capable of characterizing the exploration-exploitation dynamics throughout the optimization process.

### 3.6. Exploration and Exploitation Measurement

The structural differences among the benchmark landscapes induce distinct collective behaviors in swarm-based metaheuristics. These variations affect the spatial distribution of agents and the exploration-exploitation dynamics during the search process. Characterizing such processes requires quantitative indicators that are sensitive to the population evolution of the swarm. Consequently, metrics aimed at quantifying population diversity and analyzing search dynamics in RSA and DLO were employed.

Dimensional diversity was used as an indicator of population dispersion within the search space due to its widespread use in the analysis of the exploration–exploitation balance in swarm-based metaheuristics [5,9]. This metric makes it possible to characterize the spatial evolution of the swarm throughout the optimization process. High diversity levels reflect a predominance of exploratory behavior, whereas sustained decreases indicate exploitative intensification and population convergence.

For a dimension *j*, dimensional diversity is defined as:

(22) $${\mathrm{Div}}_{j}=\frac{1}{n}\sum_{i=1}^{n}\left|\mathrm{median}\left(x^{j}\right)-x_{i}^{j}\right|$$

where *n* represents the total number of agents, $x_{i}^{j}$ corresponds to the value of agent *i* in dimension *j*, and $\mathrm{median}(x^{j})$ denotes the population median of that dimension.

The global diversity of the swarm is obtained by averaging the diversity values computed across all dimensions of the problem:

(23) $$\mathrm{Div}=\frac{1}{m}\sum_{j=1}^{m}{\mathrm{Div}}_{j}$$

where *m* represents the dimensionality of the search space.

Based on the global diversity, relative exploration and exploitation indicators were derived. The exploration percentage quantifies the level of population dispersion relative to the maximum observable diversity, ${\mathrm{Div}}_{max}$. Complementarily, the exploitation percentage represents the degree of population concentration during the search process. Both indicators were defined as follows:

(24) $$\mathrm{XPL}\%=\left(\frac{\mathrm{Div}}{{\mathrm{Div}}_{max}}\right)\times 100$$

(25) $$\mathrm{XPT}\%=\left(\frac{|\mathrm{Div}\;-{\mathrm{Div}}_{max}|}{{\mathrm{Div}}_{max}}\right)\times 100$$

High values of XPL% indicate a predominance of exploratory behavior associated with high population dispersion, whereas high values of XPT% reflect exploitative behavior and swarm convergence processes.

### 3.7. Characterization of Exploration–Exploitation Dynamics

The exploration–exploitation dynamics were analyzed through the temporal evolution of population diversity during the optimization process. For each benchmark function, algorithm, and independent run *r*, the global diversity was recorded at every iteration *t*. A total of 30 independent runs were performed for each algorithm–function combination in order to capture the stochastic variability inherent to the search process.

To analyze the swarm dispersion dynamics, the global diversity of each run *r* was normalized using an independent min-max transformation. The intra-run temporal structure was preserved, and diversity normalization ensured comparability under a common scale:

(26) $$D_{norm_{\left(r\right)}}^{\left(t\right)}=\frac{D_{\left(r\right)}^{\left(t\right)}-D_{min_{\left(r\right)}}}{D_{max_{\left(r\right)}}-D_{min_{\left(r\right)}}}$$

where $D_{\left(r\right)}^{\left(t\right)}$ represents the global swarm diversity at iteration *t* of run *r*. the terms $D_{min_{\left(r\right)}}$ and $D_{max_{\left(r\right)}}$ represent, respectively, the minimum and maximum diversity values observed during run *r*. The normalization constrains diversity to the interval [0,1], while preserving the temporal dynamics of the swarm.

To quantify the progressive contraction of the swarm, the relative loss of normalized diversity was defined as the complement of the normalized diversity:

(27) $$L_{\left(r\right)}^{\left(t\right)}=1-D_{norm_{\left(r\right)}}^{\left(t\right)}$$

where $L_{\left(r\right)}^{\left(t\right)}$ represents the relative loss of normalized diversity of the swarm at iteration *t* of run *r*. Values close to zero indicate high population dispersion within the swarm. Values close to one indicate a greater loss of dispersion and a more concentrated population during the search process.

The exploration–exploitation dynamics were characterized through the first threshold-crossing iteration over the relative loss of normalized diversity. The threshold τ was defined within the domain [0,1], corresponding to the variation interval of $L_{\left(r\right)}^{\left(t\right)}$. For each independent run *r*, the crossing iteration was defined as the first iteration at which the relative loss of normalized diversity reached or exceeded the threshold. Formally, it was defined as:

(28) $$t_{\tau }^{\left(r\right)}=\min\left\{t|L_{\left(r\right)}^{\left(t\right)}⩾\tau \right\}$$

To analyze the temporal evolution of the relative loss of diversity, multiple referenced levels were employed over the normalized interval [0, 1]. These levels made it possible to identify different threshold-crossing events during the optimization process. The use of multiple thresholds provided temporal descriptors that are comparable across runs, algorithms, and benchmark functions.

Three threshold levels, τ∈{0.50,0.75,0.90}, were defined over the normalized relative diversity loss interval. These levels made it possible to identify different threshold-crossing events during the optimization process. The use of multiple thresholds provided temporal descriptors that are comparable across runs, algorithms, and benchmark functions.

For each threshold τ, the first-occurrence iteration $t_{\tau }^{\left(r\right)}$ was identified in each independent run. Subsequently, the distribution of $t_{\tau }^{\left(r\right)}$ was summarized using the median and the interquartile range $\left[Q_{1},Q_{3}\right]$. The median characterized the typical iteration at which the swarm reached each level of relative loss of normalized diversity. Lower values indicated earlier transitions from predominantly exploratory states toward more exploitative regimes. The interquartile range quantified the stochastic variability of these transitions and enabled the assessment of the temporal consistency of the exploration–exploitation dynamics across runs.

### 3.8. Complementary Characterization of the Search Process Using Dynamic Indicators

The temporal dynamics of the swarm were characterized using the relative loss of population diversity. This characterization was complemented with indicators associated with the spatial distribution of the agents and the progress of the optimization process. The swarm radius and the normalized fitness improvement fraction were incorporated as additional metrics of the collective dynamics. Each indicator described a specific dimension of the swarm’s behavior during the search process. An integrated characterization was obtained through the joint analysis of these metrics throughout the evolution of the swarm. The swarm radius was used to characterize the spatial configuration of the agents during optimization. For each independent run *r* and iteration *t*, this metric was defined as the mean Euclidean distance between the agents and the swarm centroid:

(29) $$R_{\left(r\right)}^{\left(t\right)}=\frac{1}{n}\sum_{i=1}^{n}{∥x_{i_{\left(r\right)}}^{\left(t\right)}-c_{\left(r\right)}^{\left(t\right)}∥}_{2}$$

where *n* denotes the number of agents. The term $x_{i_{\left(r\right)}}^{\left(t\right)}$ corresponds to the position of agent *i* at iteration *t* of run *r*. The vector $c_{\left(r\right)}^{\left(t\right)}$ denotes the swarm centroid at the same iteration. The centroid was defined as the mean position of all agents in the swarm.

High values of the swarm radius indicate greater spatial separation among the swarm agents. Low values reflect a more spatially concentrated swarm during the optimization process. This metric complements the information provided by the relative loss of population diversity.

The progress of the optimization process was quantified using the normalized fitness improvement fraction. For each independent run *r* and iteration *t*, this metric was defined as follows:

(30) $$P_{\left(r\right)}^{\left(t\right)}=\frac{f_{0}^{\left(r\right)}-f_{best_{\left(r\right)}}^{\left(t\right)}}{f_{0}^{\left(r\right)}-f_{best_{\left(r\right)}}^{\left(T\right)}}$$

where $f_{0}^{\left(r\right)}$ denotes the best fitness recorded at the beginning of run *r*. The term $f_{best_{\left(r\right)}}^{\left(t\right)}$ corresponds to the best fitness achieved up to iteration *t* of run *r*. The value $f_{best_{\left(r\right)}}^{\left(T\right)}$ represents the best fitness attained at the end of run *r*.

For a given run *r*, values of $P_{\left(r\right)}^{\left(t\right)}$ close to one indicate that the swarm has achieved a large proportion of the total fitness improvement recorded during the run. Values close to zero indicate limited relative progress with respect to the total improvement attained by the end of the run. This metric describes the evolution of the swarm’s performance throughout the optimization process.

For each normalized relative diversity loss threshold τ, both metrics were evaluated at the first-occurrence iteration $t_{\tau }^{\left(r\right)}$. This iteration was determined using the threshold-crossing criterion defined previously. The values of the swarm radius and the normalized fitness improvement fraction at iteration $t_{\tau }^{\left(r\right)}$ were used to characterize the swarm configuration at the corresponding threshold-crossing event.

The metrics were computed independently for each run. Subsequently, their distributions were summarized using the median and the interquartile range $\left[Q_{1},Q_{3}\right]$. This procedure preserved the temporal variability associated with each search trajectory. These summary statistics describe the central tendency and variability of the swarm radius and the normalized fitness improvement fraction at each threshold-crossing event.

### 3.9. Experimental Settings

Computational experiments were conducted using the RSA, DLO, and PSO metaheuristics. RSA and DLO were selected as the case studies, whereas PSO was included as a widely used reference algorithm in the literature. A population of 30 agents was used for both metaheuristics. The population size was kept constant across all benchmark functions and evaluated dimensions.

The benchmark problem set consisted of the Sphere, Rosenbrock, Rastrigin, Ackley, and Griewank functions. Each function was evaluated in dimensions n∈{30,50,100}. The analytical expressions and search domains considered are presented in Table 1.

To capture the stochastic variability inherent to the search process, RSA, DLO and PSO were executed using 30 independent runs for each function–dimension combination.

The maximum number of iterations was determined experimentally for each benchmark function, ensuring that the search trajectories reached a stable convergence phase accompanied by a marked reduction in population diversity. Under this criterion, 250 iterations were used for the Sphere, Rosenbrock, and Griewank functions. For Ackley, 300 iterations were employed, whereas 350 iterations were used for Rastrigin.

The same experimental configuration was applied to both metaheuristics across all evaluated scenarios.

## 4. Results

Table 2, Table 3 and Table 4 present the optimization results obtained by RSA, DLO, and PSO on the benchmark functions evaluated in 30, 50, and 100 dimensions.

For Sphere, Ackley, and Griewank, consistent patterns were observed across the three dimensions. RSA reached the global optimum in all 30 independent runs for each dimension. DLO achieved mean objective values below $3\times 10^{-2}$ for all three functions. In PSO, the mean objective values increased as the dimensionality increased. The standard deviation was zero or nearly zero for RSA. DLO and PSO exhibited greater variability across runs.

For Rosenbrock, RSA achieved mean objective values of 29, 49, and 99 for 30, 50, and 100 dimensions, respectively. DLO obtained mean values of 27.4, 48.0, and 98.2 for the same dimensions. In PSO, the mean objective values were $3.15\times 10^{2}$, $4.48\times 10^{3}$, and $6.04\times 10^{4}$. The standard deviation remained zero for RSA and increased for DLO and PSO.

For Rastrigin, RSA reached the global optimum in all three evaluated dimensions. DLO obtained a mean objective value of 5.32 in 30 dimensions. For 50 and 100 dimensions, the mean objective values were $1.09\times 10^{-2}$ and $1.47\times 10^{-2}$, respectively. In PSO, the mean objective values increased from 34.0 to 160.0 as the dimensionality increased.

**Figure 3:**
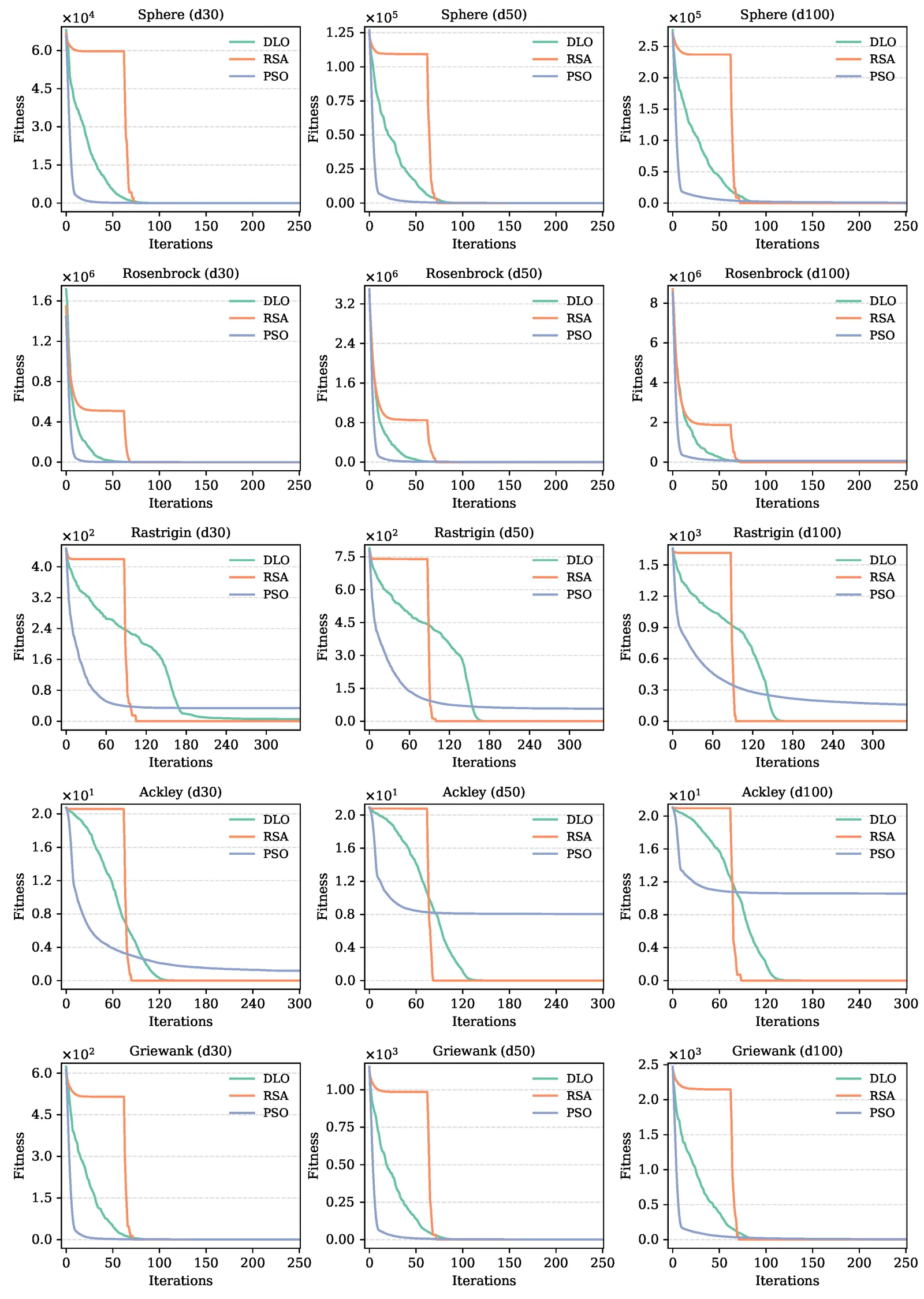
Average convergence curves of RSA, DLO and PSO across Benchmark Functions and Dimensions.

The mean convergence curves, computed from the 30 independent runs of each metaheuristic, are presented in Figure 3. Distinct convergence patterns were observed depending on the benchmark function.

For Sphere, Ackley, and Griewank, the largest reduction in the fitness value for RSA occurred during the first iterations. Thereafter, the convergence curves remained nearly stable until the end of the optimization process. This pattern was consistently observed across all three evaluated dimensions.

For the same functions, DLO distributed the reduction in the fitness value over a larger fraction of the iteration budget. Subsequently, the convergence curves reached a stabilization phase. This pattern was consistently observed across all three evaluated dimensions.

For Sphere, Ackley, and Griewank, PSO also distributed the reduction in the fitness value over a larger fraction of the optimization process. Subsequently, the convergence curves reached a stabilization phase. This behavior was consistently observed across all three evaluated dimensions.

For Rosenbrock, all three metaheuristics concentrated the largest reduction in the fitness value during the first iterations. Thereafter, the convergence curves evolved toward a stabilization phase. This behavior was observed across all three dimensions.

For Rastrigin, RSA concentrated the reduction in the fitness value during the first iterations for all three evaluated dimensions. In DLO, the reduction in the fitness value was distributed over a larger fraction of the iteration budget in 30 dimensions than in 50 and 100 dimensions. In PSO, the convergence curves exhibited a continuous reduction in the fitness value followed by a stabilization phase across all three dimensions.

**Figure 4:**
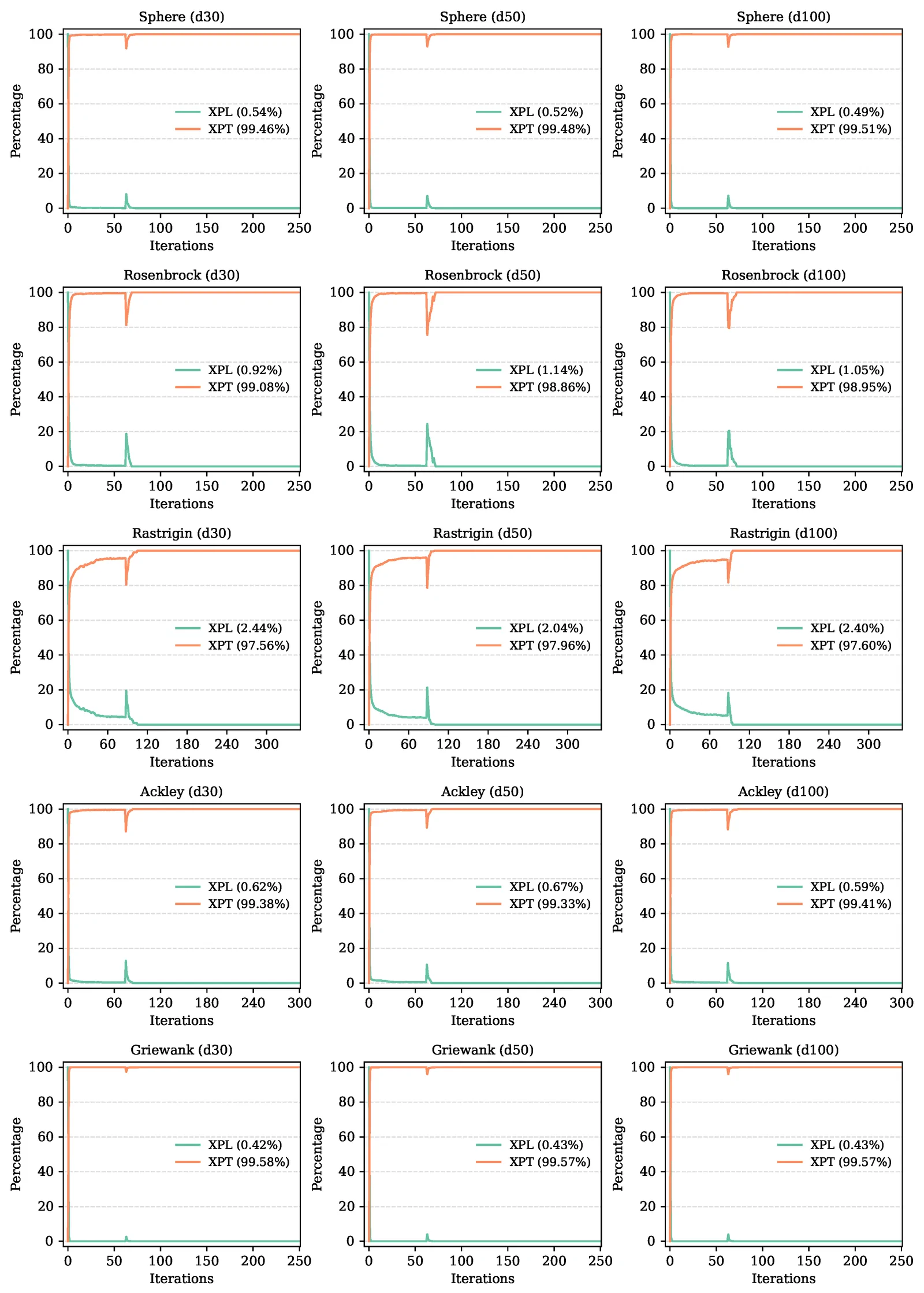
Exploration and Exploitation of RSA Across Benchmark Functions and Dimensions.

**Figure 5:**
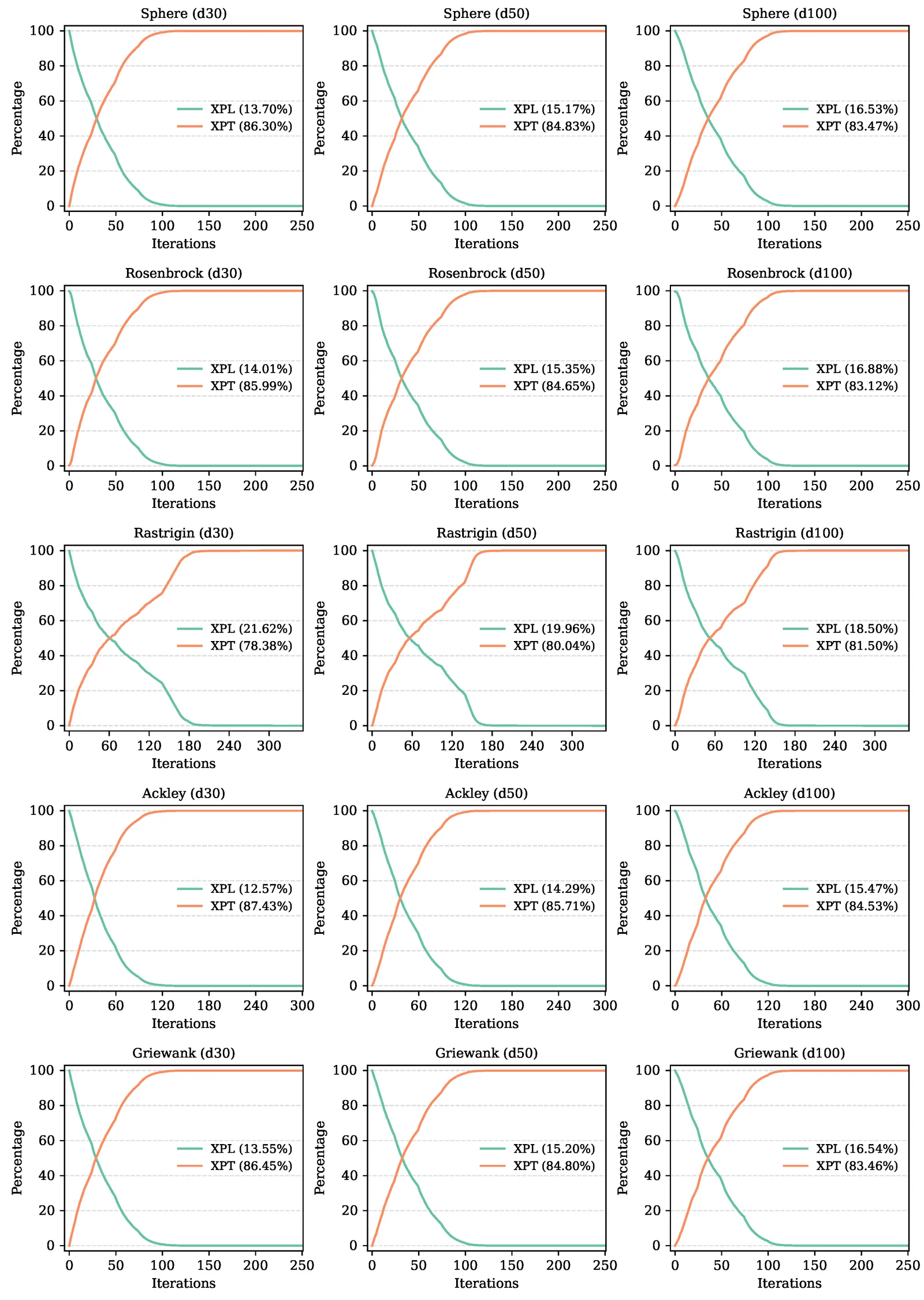
Exploration and Exploitation of DLO Across Benchmark Functions and Dimensions.

**Figure 6:**
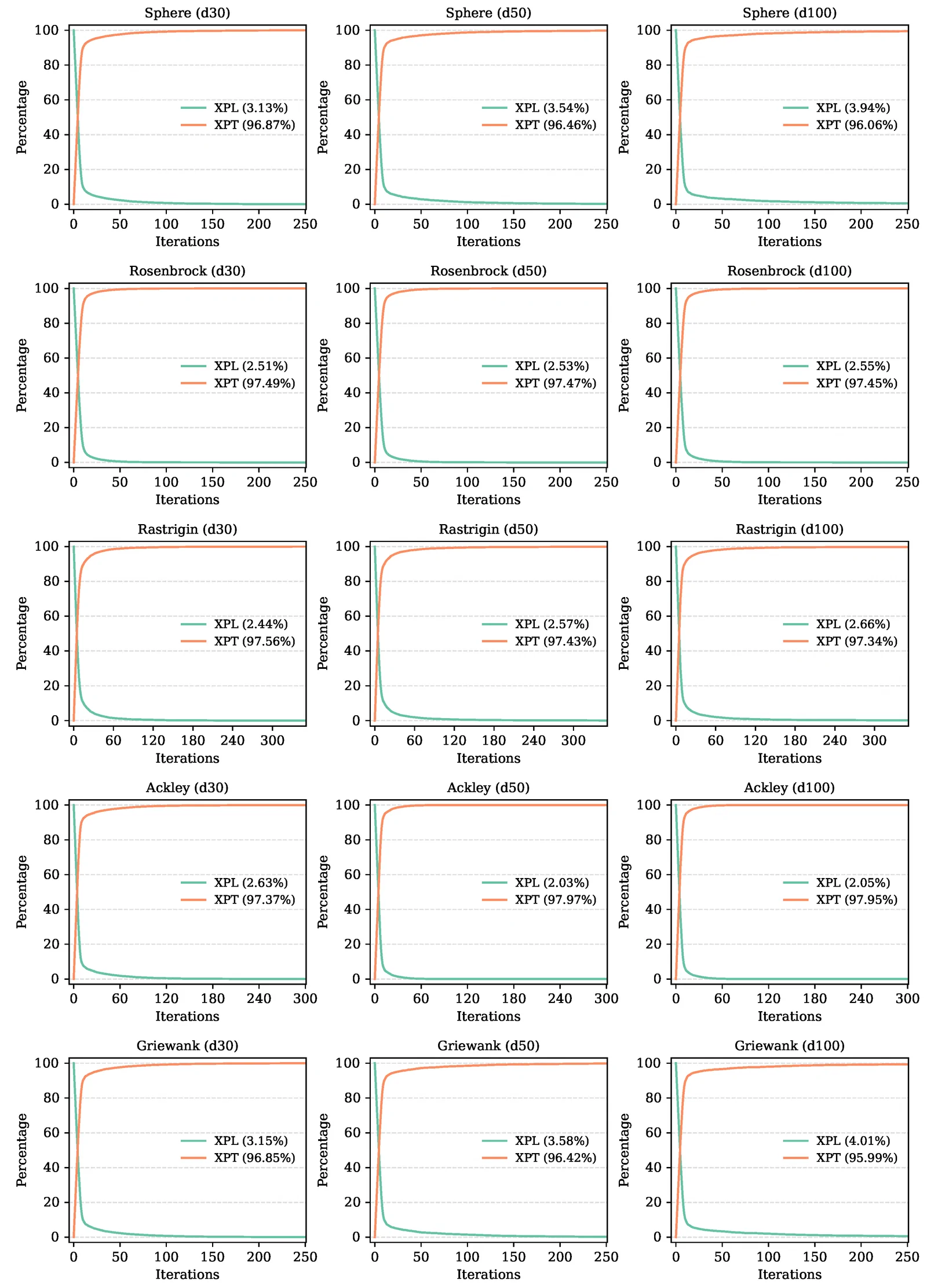
Exploration and Exploitation of PSO Across Benchmark Functions and Dimensions.

The mean exploration and exploitation curves, computed from the 30 independent runs, are presented in Figure 4, Figure 5 and Figure 6. Distinct patterns were observed among the three metaheuristics.

For RSA, exploitation predominated throughout the optimization process across the five benchmark functions and the three evaluated dimensions. The exploration percentages ranged from 0.42% to 2.44%, whereas the exploitation percentages ranged from 97.56% to 99.58%. Both components exhibited only minor variations throughout the iterative process.

For DLO, exploitation also predominated throughout the optimization process. The exploration percentages ranged from 12.57% to 21.62%, whereas the exploitation percentages remained between 78.38% and 87.43%. Both components maintained a stable temporal behavior across all benchmark functions and evaluated dimensions.

For PSO, exploitation likewise predominated throughout the optimization process. The exploration percentages ranged from 2.03% to 4.01%, whereas the exploitation percentages remained between 95.99% and 97.97%. The corresponding curves exhibited only minor variations throughout the iterative process across the five benchmark functions and the three evaluated dimensions.

The differences among the metaheuristics remained stable throughout the optimization process. RSA exhibited the lowest exploration percentages, DLO the highest, and PSO intermediate values across all benchmark functions and evaluated dimensions.

**Figure 7:**
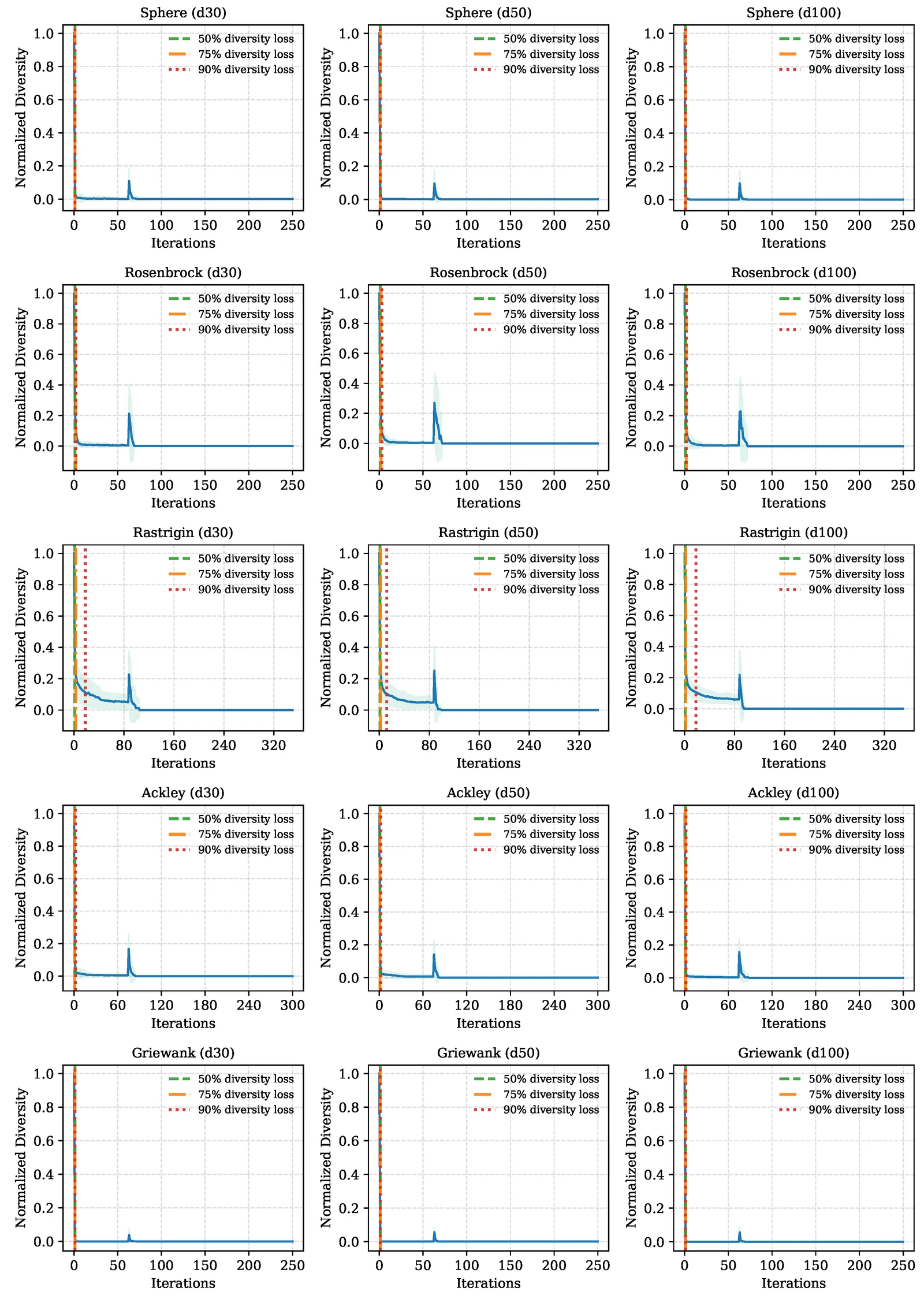
Normalized Diversity Dynamics and Diversity Loss Events in RSA.

**Figure 8:**
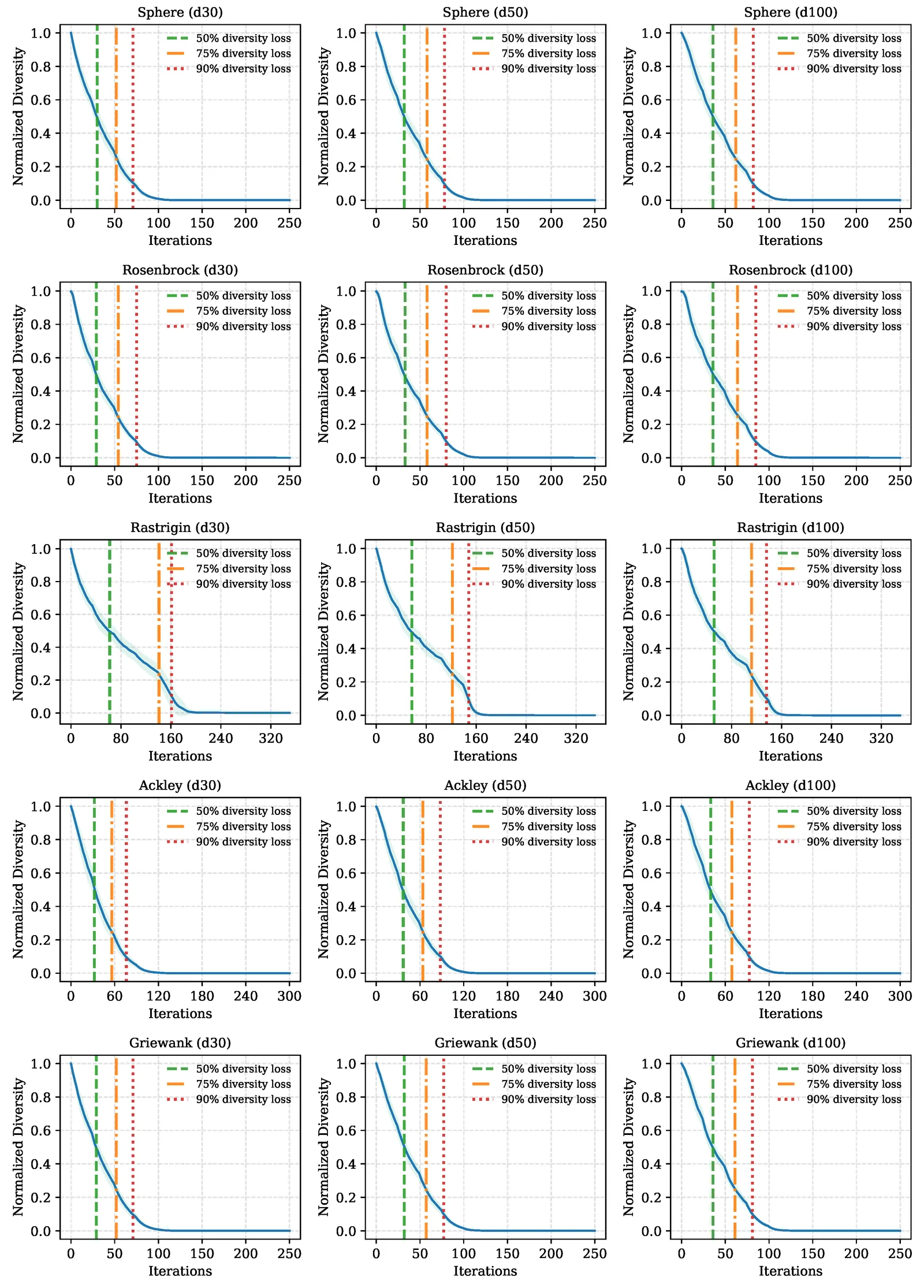
Normalized Diversity Dynamics and Diversity Loss Events in DLO.

**Figure 9:**
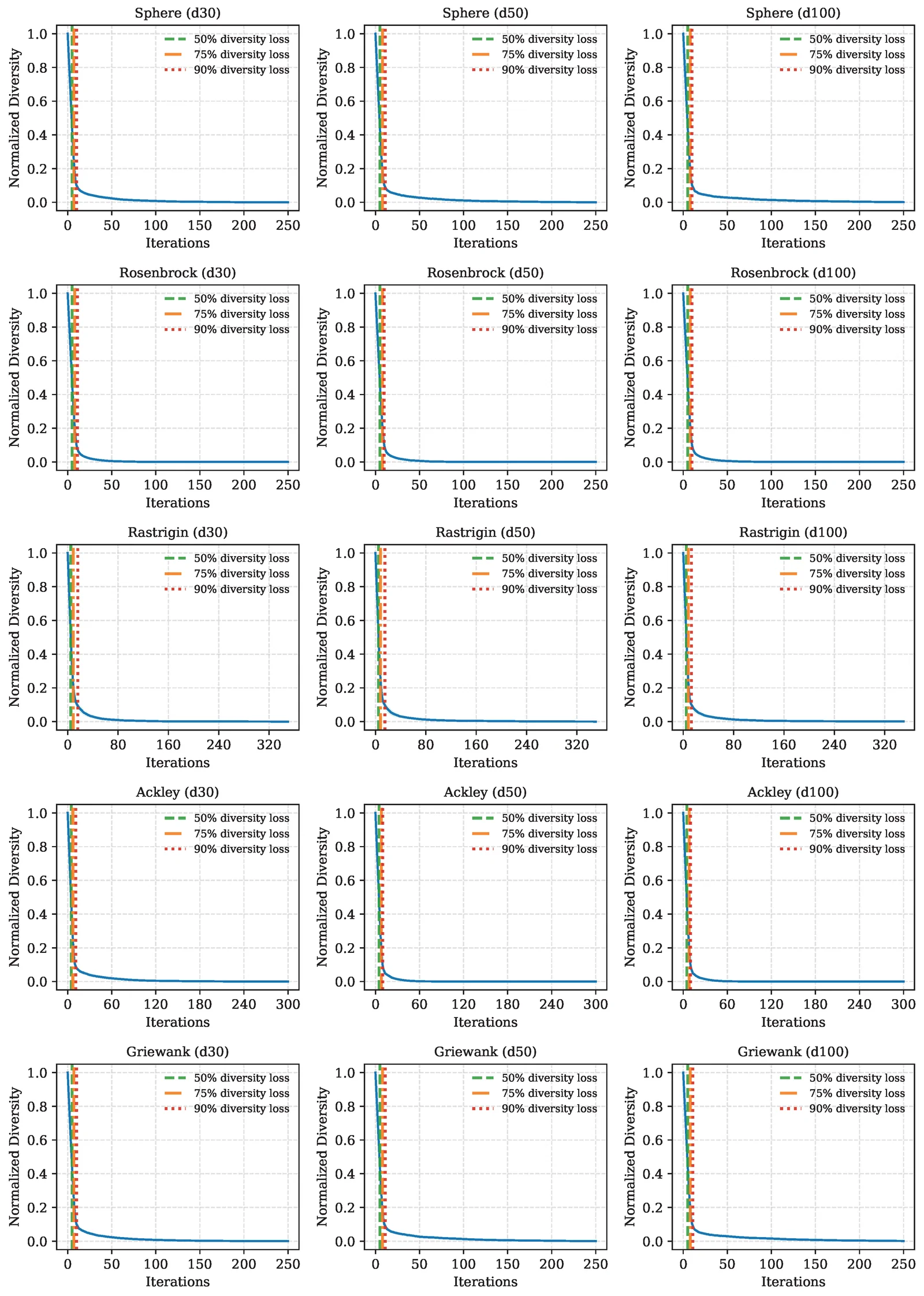
Normalized Diversity Dynamics and Diversity Loss Events in PSO.

The normalized diversity dynamics and the events associated with the operational levels of relative diversity loss are presented in Figure 7, Figure 8 and Figure 9. The medians and interquartile ranges of the first-threshold-crossing iterations are summarized in Table 5, Table 6 and Table 7.

For RSA, the normalized diversity decreased sharply during the first iterations across the five benchmark functions and the three evaluated dimensions. Thereafter, the curves remained close to their minimum values until the end of the optimization process. The differences among benchmark functions were small, although Rastrigin exhibited a slightly more gradual decrease than the remaining functions.

For DLO, the normalized diversity decreased over a larger fraction of the optimization process. The curves subsequently reached a stabilization phase for all benchmark functions. Rastrigin maintained higher normalized diversity levels for more iterations than Sphere, Rosenbrock, Ackley, and Griewank across the three evaluated dimensions.

For PSO, the normalized diversity decreased primarily during the first iterations across the five benchmark functions and the three evaluated dimensions. Thereafter, the curves remained close to their minimum values until the end of the execution.

The first-threshold-crossing iterations revealed consistent differences among the three metaheuristics. For RSA, the operational level corresponding to a 50% relative diversity loss was reached at the first iteration for all benchmark functions and evaluated dimensions. The 75% and 90% operational levels were also reached during the first iterations in most experimental configurations.

For DLO, the median first-threshold-crossing iterations ranged from 29 to 62 for the 50% operational level, from 52 to 141 for the 75% level, and from 71 to 161 for the 90% level. Rastrigin consistently exhibited the largest median first-threshold-crossing iterations, whereas Sphere, Rosenbrock, Ackley, and Griewank produced lower values across the three evaluated dimensions.

For PSO, all three operational levels were reached during the first iterations across the five benchmark functions and the three evaluated dimensions. The median first-threshold-crossing iterations were five for the 50% operational level, between seven and eight for the 75% level, and between ten and seventeen for the 90% level. Once again, Rastrigin exhibited the largest median first-threshold-crossing iterations among the evaluated benchmark functions.

The medians of the swarm radius, their interquartile ranges, and the relative contraction with respect to the initial radius are presented in Table 8, Table 9 and Table 10. The results are reported for the beginning of the optimization process, the operational levels of relative diversity loss, and the final iteration.

The initial swarm radii were similar across the three metaheuristics for all benchmark functions and evaluated dimensions. The observed differences among the algorithms were small.

For RSA, the spatial contraction was more pronounced than for the other metaheuristics. At the 50% operational level, the $R_{\tau }/R_{0}$ ratios ranged from 0.03 to 0.57. At the 75% and 90% operational levels, the ratios decreased to ranges of 0.03–0.47 and 0.03–0.30, respectively. For all benchmark functions, the final swarm radius was equal to zero.

For DLO, the largest $R_{\tau }/R_{0}$ ratios were consistently observed across the three operational levels. At the 50% operational level, the ratios ranged from 0.53 to 0.57. At the 75% level, the values ranged from 0.27 to 0.31. At the 90% level, the ratios remained between 0.11 and 0.14. The final swarm radii were zero or close to zero for all benchmark functions.

For PSO, the $R_{\tau }/R_{0}$ ratios exhibited intermediate values relative to RSA and DLO. At the 50% operational level, the ratios ranged from 0.46 to 0.50. At the 75% level, the values ranged from 0.21 to 0.26. At the 90% level, the ratios remained between 0.10 and 0.11. The final swarm radii were zero or close to zero across all experimental configurations.

All three metaheuristics exhibited a progressive reduction in the swarm radius across the operational levels considered. However, the largest differences among the algorithms were observed at the 50% and 75% operational levels, where DLO preserved the largest $R_{\tau }/R_{0}$ ratios, PSO exhibited intermediate values, and RSA produced the smallest ratios for most benchmark functions.

Table 11, Table 12 and Table 13 present the evolution of the fitness improvement fraction at the operational levels of relative diversity loss.

For RSA, the fitness improvement fraction remained close to zero across the three operational levels for Sphere, Rastrigin, Ackley, and Griewank. For Rosenbrock, a gradual increase was observed. The median reached values ranging from 0.27 to 0.32 at τ=0.90, depending on the dimensionality.

For DLO, a large proportion of the final fitness improvement had already been achieved at τ=0.50 for Sphere, Rosenbrock, and Griewank. The medians ranged from 0.73 to 0.76 for Sphere and Griewank, and from 0.93 to 0.96 for Rosenbrock. At τ=0.90, the medians reached values between 0.99 and 1.00.

For DLO, Rastrigin exhibited a more gradual evolution. The medians increased from 0.36–0.39 at τ=0.50 to 0.73–0.82 at τ=0.90 Ackley also exhibited a progressive increase. The medians ranged from 0.13 to 0.16 at τ=0.50 and from 0.62 to 0.67 at τ=0.90.

For PSO, Sphere and Griewank exhibited a pattern similar to that observed for DLO. The medians ranged from 0.71 to 0.72 at τ=0.50, from 0.88 to 0.90 at τ=0.75, and from 0.94 to 0.95 at τ=0.90.

For PSO, Rosenbrock exhibited medians ranging from 0.78 to 0.81 at τ=0.50. These medians increased to values between 0.96 and 0.97 at τ=0.90.

For PSO, Rastrigin exhibited a slower progression than Sphere, Rosenbrock, and Griewank. The medians remained between 0.34 and 0.36 at τ=0.50, between 0.45 and 0.48 at τ=0.75, and between 0.54 and 0.62 at τ=0.90.

For PSO, Ackley exhibited a gradual increase in the fitness improvement fraction. The medians ranged from 0.15 to 0.23 at τ=0.50, from 0.31 to 0.55 at τ=0.75, and from 0.48 to 0.72 at τ=0.90.

## 5. Discussion

Exploration and exploitation constitute fundamental properties of collective behavior in swarm metaheuristics, as they determine the evolution of the search process [39,40]. Their study is essential for understanding how interactions among agents influence the collective dynamics of the swarm [39].

Despite their importance, the experimental characterization of this dynamic remains a methodological challenge [41,42]. Most studies analyze it through individual indicators, which describe different properties of the search process [43].

In this context, the present study integrates normalized diversity, the mean swarm radius, and the improvement fraction as complementary dynamic indicators. Their joint analysis enables the simultaneous study of population dispersion evolution, swarm spatial organization, and optimization progress throughout the search process. Together, these indicators establish an experimental framework for studying swarm collective dynamics from complementary perspectives.

Relative diversity loss levels allow the establishment of homogeneous comparison points throughout the search process. These points define equivalent population diversity states for different metaheuristics, benchmark functions, and dimensions.

The usefulness of this criterion is evidenced by the measurements of the mean radius and improvement fraction performed at the operational levels of 50%, 75%, and 90% relative diversity loss. Under these same levels, RSA presents systematically smaller mean radii than DLO, whereas PSO exhibits intermediate values. Likewise, for the same operational level, DLO and PSO achieve different improvement fractions across Sphere, Rosenbrock, Ackley, and Rastrigin.

These observations suggest that relative diversity loss constitutes an appropriate criterion for synchronizing the comparison of different search processes. At the same time, it provides a common reference framework for analyzing the evolution of swarm collective behavior [44].

The evolution of population dispersion, swarm spatial organization, and optimization progress exhibits different temporal trajectories throughout the search process.

This difference is observed across multiple experimental configurations. In DLO, similar spatial contraction ratios at the 50% and 75% operational levels are associated with clearly different improvement fractions among Sphere, Ackley, and Rastrigin. A similar behavior is identified in PSO, where comparable reductions in mean radius coexist with optimization progress of different magnitudes depending on the benchmark function.

These observations indicate that the evolution of population dispersion, spatial organization, and optimization progress responds to complementary processes of collective behavior. Consequently, their joint analysis promotes a more comprehensive interpretation of the transitions associated with the balance between exploration and exploitation [45].

The differences observed among the benchmark functions were consistent with the mathematical properties considered in the experimental design [46,47]. Modality, separability, ruggedness, and conditioning defined search scenarios with different exploration and exploitation demands [28,47].

This influence was reflected in the evolution of the dynamic indicators. Sphere exhibited early reductions in population diversity, rapid swarm spatial contraction, and a high improvement fraction from the first operational levels. In Rosenbrock, spatial reorganization occurred early. However, an important proportion of the improvement with respect to the final solution was achieved at later levels of relative diversity loss.

The multimodal functions also exhibited differentiated behaviors [26,35]. Rastrigin maintained high diversity levels over a larger number of iterations [5] and presented a more gradual evolution of the improvement fraction. Ackley showed a relatively early reduction in diversity, although an important proportion of the improvement with respect to the final solution was achieved at later levels of relative diversity loss. In Griewank, the improvement fraction reached high values from the first operational levels, despite its multimodal nature.

These differences also depended on the search mechanisms of each metaheuristic. RSA concentrated swarm reorganization at early stages. DLO distributed this process over a larger proportion of the iterative budget. PSO exhibited an intermediate trajectory between both behaviors. These trends were preserved across the three evaluated dimensions.

Overall, these observations indicate that the evolution of swarm collective behavior was simultaneously associated with the mathematical properties of the landscape and the search mechanisms of each metaheuristic. Furthermore, they support the use of multiple dynamic indicators to experimentally analyze this interaction under different optimization scenarios.

Increasing dimensionality modified the values reached by the dynamic indicators and the number of iterations required to achieve the different operational levels of relative diversity loss. However, the qualitative evolution patterns were preserved across dimensions 30, 50, and 100.

This consistency was observed in the three evaluated metaheuristics. RSA maintained early swarm reorganization across all dimensions. DLO preserved a more gradual evolution of diversity, mean radius, and improvement fraction. PSO again exhibited an intermediate behavior. Similarly, Rastrigin continued to show the most prolonged transitions among the benchmark functions, whereas Sphere maintained the earliest ones.

The preservation of these patterns under different dimensions strengthens the robustness of the experimental observations. It also suggests that the proposed methodology enables the consistent study of swarm collective dynamics in problems with different dimensional scales.

In several swarm metaheuristics, exploration and exploitation are associated with search mechanisms defined during algorithm design [48,49]. In RSA and DLO, these mechanisms are explicitly organized into two consecutive stages. The first stage incorporates operators oriented toward exploration. The second stage incorporates operators oriented toward exploitation [18,19].

The experimental evidence obtained showed that the earliest transitions observed in collective dynamics for RSA and DLO occurred before the transitions between stages defined by these search mechanisms. In RSA, the operational level corresponding to 50% relative diversity loss was reached at the first iteration for all benchmark functions and dimensions. The 75% and 90% levels were also reached during the first iterations. In DLO, the transition was more gradual. However, the three operational levels were also reached before half of the iterative budget.

These observations show that the temporal organization of search mechanisms does not necessarily describe the temporal evolution of swarm collective behavior. Consequently, experimental evaluation based on dynamic indicators enables the assessment of the correspondence between algorithmic design and the collective behavior observed during optimization [50].

## 6. Conclusions

This work addressed the experimental validation of emergent behaviors in bio-inspired metaheuristics, with emphasis on the exploration-exploitation balance. A gap in the literature was identified, associated with the limited validation of search dynamics and the predominant use of final performance metrics, which do not capture the temporal evolution of the search process.

In this context, a reproducible methodology based on dynamic population diversity indicators was proposed and validated. Its applicability was demonstrated through the RSA and DLO algorithms by contrasting the observed dynamics with their design claims. The obtained results challenge the direct inference of emergent behaviors from metaphors or conceptual descriptions.

RSA exhibited a highly exploitative dynamic from the first iterations, accompanied by an early collapse of population diversity, providing evidence of the effective absence of the exploratory phase described in its conceptual design. In contrast, DLO exhibited a more progressive biphasic dynamic, with greater diversity preservation during a significant portion of the search process. However, this exploratory phase did not extend to half of the iterative budget. Additionally, the structure of the search landscape was found to modulate the expression of the internal mechanisms in both algorithms.

The limitations of the study are related to the benchmark function set considered, which restricts generalization to more complex scenarios or real-world applications. Likewise, the exploration and exploitation metrics constitute indirect approximations of population behavior and may therefore overlook relevant local dynamics.

Future research should extend this analysis to real-world problems and incorporate complementary measures of population structure. It is also recommended to explore adaptive mechanisms that explicitly regulate exploration-exploitation dynamics according to the state of the system.

Taken together, this study contributes to the formulation of an evaluation framework based on dynamic diversity indicators, providing evidence that emergent behaviors cannot be inferred exclusively from algorithmic design. This approach enables a more rigorous and operational characterization of bio-inspired metaheuristics based on empirical evidence of system behavior.

## Figures and Tables

**Table 1:** Benchmark Problems Used in the Experimental Evaluation.

Name	Function	Dimensions	Range
Sphere	$\sum_{i=1}^{n}x_{i}^{2}$	{30,50,100}	${\left[-100,100\right]}^{n}$
Rosenbrock	$\sum_{i=1}^{n-1}\left[100{\left(x_{i+1}-x_{i}^{2}\right)}^{2}+{\left(x_{i}-1\right)}^{2}\right]$	{30,50,100}	${\left[-5,10\right]}^{n}$
Rastrigin	$10n+\sum_{i=1}^{n}\left[x_{i}^{2}-10\cos\left(2\pi x_{i}\right)\right]$	{30,50,100}	${\left[-5.12,5.12\right]}^{n}$
Ackley	$-20\exp\left(-0.2\sqrt{\frac{1}{n}\sum_{i=1}^{n}x_{i}^{2}}\right)-\exp\left(\frac{1}{n}\sum_{i=1}^{n}\cos\left(2\pi x_{i}\right)\right)+20+e$	{30,50,100}	${\left[-32.786,32.786\right]}^{n}$
Griewank	$1+\frac{1}{4000}\sum_{i=1}^{n}x_{i}^{2}-\prod_{i=1}^{n}\cos\left(\frac{x_{i}}{\sqrt{i}}\right)$	{30,50,100}	${\left[-600,600\right]}^{n}$

**Table 2:** Statistical results obtained for the benchmark function (dimension 30).

Metaheuristics	Metrics	Sphere	Rosenbrock	Rastrigin	Ackley	Griewank
RSA	Best	0.00	2.90 × 10^1^	0.00	4.44 × 10^−16^	0.00
	Average	0.00	2.90 × 10^1^	0.00	4.44 × 10^−16^	0.00
	STD	0.00	0.00	0.00	5.01 × 10^−32^	0.00
	%XPL	0.54	0.92	2.44	0.62	0.42
	%XPT	99.46	99.08	97.56	99.38	99.58
DLO	Best	3.58 × 10^−6^	2.66 × 10^1^	5.86 × 10^−5^	5.47 × 10^−5^	2.28 × 10^−6^
	Average	2.49 × 10^−5^	2.74 × 10^1^	5.32	1.37 × 10^−4^	8.25 × 10^−5^
	STD	2.14 × 10^−5^	5.40 × 10^−1^	2.57 × 10^1^	5.54 × 10^−5^	1.34 × 10^−4^
	%XPL	13.70	14.01	21.62	12.57	13.55
	%XPT	86.30	85.99	78.38	87.43	86.45
PSO	Best	2.14 × 10^−3^	1.27 × 10^2^	1.39 × 10^1^	1.01 × 10^−2^	8.51 × 10^−3^
	Average	5.10 × 10^−2^	3.15 × 10^2^	3.40 × 10^1^	1.18	7.53 × 10^−2^
	STD	4.92 × 10^−2^	1.47 × 10^2^	1.09 × 10^1^	9.26 × 10^−1^	7.79 × 10^−2^
	%XPL	3.13	2.51	2.44	2.63	3.15
	%XPT	96.87	97.49	97.56	97.37	96.85

**Table 3:** Statistical results obtained for the benchmark function (dimension 50).

Metaheuristics	Metrics	Sphere	Rosenbrock	Rastrigin	Ackley	Griewank
RSA	Best	0.00	4.90 × 10^1^	0.00	4.44 × 10^−16^	0.00
	Average	0.00	4.90 × 10^1^	0.00	4.44 × 10^−16^	0.00
	STD	0.00	0.00	0.00	5.01 × 10^−32^	0.00
	%XPL	0.52	1.14	2.04	0.67	0.43
	%XPT	99.48	98.86	97.96	99.33	99.57
DLO	Best	3.44 × 10^−4^	4.70 × 10^1^	1.14 × 10^−3^	3.76 × 10^−4^	4.28 × 10^−4^
	Average	1.47 × 10^−3^	4.80 × 10^1^	1.09 × 10^−2^	1.16 × 10^−3^	2.01 × 10^−3^
	STD	8.59 × 10^−4^	3.88 × 10^−1^	1.40 × 10^−2^	5.22 × 10^−4^	1.78 × 10^−3^
	%XPL	15.17	15.35	19.96	14.29	15.20
	%XPT	84.83	84.65	80.04	85.71	84.80
PSO	Best	1.47	1.17 × 10^3^	2.95 × 10^1^	5.81	9.81 × 10^−1^
	Average	1.07 × 10^1^	4.48 × 10^3^	5.68 × 10^1^	8.04	1.11
	STD	9.75	2.47 × 10^3^	1.37 × 10^1^	1.01	1.03 × 10^−1^
	%XPL	3.54	2.53	2.57	2.03	3.58
	%XPT	96.46	97.47	97.43	97.97	96.42

**Table 4:** Statistical results obtained for the benchmark function (dimension 100).

Metaheuristics	Metrics	Sphere	Rosenbrock	Rastrigin	Ackley	Griewank
RSA	Best	0.00	9.90 × 10^1^	0.00	4.44 × 10^−16^	0.00
	Average	0.00	9.90 × 10^1^	0.00	4.44 × 10^−16^	0.00
	STD	0.00	0.00	0.00	5.01 × 10^−32^	0.00
	%XPL	0.49	1.05	2.40	0.59	0.43
	%XPT	99.51	98.95	97.60	99.41	99.57
DLO	Best	1.23 × 10^−2^	9.77 × 10^1^	1.23 × 10^−3^	2.87 × 10^−3^	5.14 × 10^−3^
	Average	2.75 × 10^−2^	9.82 × 10^1^	1.47 × 10^−2^	5.23 × 10^−3^	2.02 × 10^−2^
	STD	1.01 × 10^−2^	2.13 × 10^−1^	3.82 × 10^−2^	1.59 × 10^−3^	1.22 × 10^−2^
	%XPL	16.53	16.88	18.50	15.47	16.54
	%XPT	83.47	83.12	81.50	84.53	83.46
PSO	Best	2.55 × 10^2^	3.66 × 10^4^	1.16 × 10^2^	9.31	3.61
	Average	6.01 × 10^2^	6.04 × 10^4^	1.60 × 10^2^	1.06 × 10^1^	5.62
	STD	2.46 × 10^2^	1.64 × 10^4^	2.52 × 10^1^	7.29 × 10^−1^	1.37
	%XPL	3.94	2.55	2.66	2.05	4.01
	%XPT	96.06	97.45	97.34	97.95	95.99

**Table 5:** Median and Interquartile Range of First Threshold-Crossing Iterations Associated with Diversity Loss Levels in 30 Dimensions.

Metaheuristics	Threshold	Sphere	Rosenbrock	Rastrigin	Ackley	Griewank
RSA	τ=0.50	1 [1–1]	1 [1–1]	1 [1–1]	1 [1–1]	1 [1–1]
	τ=0.75	1 [1–1]	2 [2–2]	3 [2–6]	1 [1–1]	1 [1–1]
	τ=0.90	1 [1–1]	2 [2–3]	18 [5–40]	2 [1–2]	1 [1–1]
DLO	τ=0.50	30 [27–32]	29 [28–30]	62 [53–70]	32 [31–36]	29 [28–31]
	τ=0.75	52 [51–55]	54 [53–56]	141 [127–146]	56 [53–60]	52 [51–53]
	τ=0.90	71 [69–73]	75 [72–76]	161 [154–164]	76 [73–78]	71 [68–73]
PSO	τ=0.50	5 [5–5]	5 [5–5]	5 [5–5]	5 [5–5]	5 [5–5]
	τ=0.75	7 [7–8]	8 [8–8]	9 [8–9]	7 [7–8]	7 [7–8]
	τ=0.90	10 [10–11]	11 [10–11]	16 [14–17]	11 [10–11]	10 [10–11]

**Table 6:** Median and Interquartile Range of First Threshold-Crossing Iterations Associated with Diversity Loss Levels in 50 Dimensions.

Metaheuristics	Threshold	Sphere	Rosenbrock	Rastrigin	Ackley	Griewank
RSA	τ=0.50	1 [1–1]	1 [1–1]	1 [1–1]	1 [1–1]	1 [1–1]
	τ=0.75	1 [1–1]	2 [2–2]	2 [2–3]	1 [1–1]	1 [1–1]
	τ=0.90	1 [1–1]	3 [2–3]	12 [4–24]	2 [1–2]	1 [1–1]
DLO	τ=0.50	32 [31–34]	33 [29–35]	57 [53–63]	37 [33–39]	32 [31–34]
	τ=0.75	58 [54–61]	58 [56–60]	122 [116–130]	64 [61–66]	57 [56–58]
	τ=0.90	78 [76–80]	80 [79–81]	148 [144–151]	88 [84–90]	77 [76–78]
PSO	τ=0.50	5 [5–5]	5 [5–5]	5 [5–5]	5 [5–5]	5 [5–5]
	τ=0.75	8 [7–8]	8 [8–8]	8 [8–9]	8 [8–8]	8 [7–8]
	τ=0.90	11 [10–11]	10 [10–11]	15 [13–16]	10 [10–10]	11 [10–11]

**Table 7:** Median and Interquartile Range of First Threshold-Crossing Iterations Associated with Diversity Loss Levels in 100 Dimensions.

Metaheuristics	Threshold	Sphere	Rosenbrock	Rastrigin	Ackley	Griewank
RSA	τ=0.50	1 [1–1]	1 [1–1]	1 [1–1]	1 [1–1]	1 [1–1]
	τ=0.75	1 [1–1]	2 [2–2]	2 [2–3]	1 [1–1]	1 [1–1]
	τ=0.90	1 [1–1]	2 [2–3]	18 [9–29]	2 [1–2]	1 [1–1]
DLO	τ=0.50	36 [33–40]	36 [33–41]	52 [47–62]	40 [38–42]	36 [33–38]
	τ=0.75	62 [60–65]	64 [61–68]	112 [109–115]	69 [66–72]	61 [59–64]
	τ=0.90	82 [80–83]	85 [83–86]	136 [131–140]	93 [91–95]	81 [80–83]
PSO	τ=0.50	5 [5–5]	5 [5–5]	5 [5–5]	5 [5–5]	5 [5–5]
	τ=0.75	8 [8–8]	8 [8–8]	8 [8–8]	8 [8–8]	8 [8–8]
	τ=0.90	10 [10–11]	10 [10–11]	13 [13–14]	10 [10–10]	11 [11–11]

**Table 8:** Swarm Radius and Relative Contraction at Operational Diversity Loss Levels (30 Dimensions).

Metaheuristics	Threshold	Sphere	Rosenbrock	Rastrigin	Ackley	Griewank
RSA	Initial	308.66 [304.87–312.05] 1.00	23.19 [22.98–23.36] 1.00	15.82 [15.68–15.99] 1.00	101.41 [100.21–102.90] 1.00	1859.03 [1837.23–1877.25] 1.00
	τ=0.50	24.81 [16.84–31.19] 0.08	9.96 [7.85–10.87] 0.42	8.99 [7.79–10.00] 0.57	20.07 [12.27–27.43] 0.20	52.22 [47.99–87.85] 0.03
	τ=0.75	24.81 [16.84–31.19] 0.08	5.85 [4.38–7.10] 0.25	6.86 [5.58–7.86] 0.43	20.07 [12.27–27.43] 0.20	52.22 [47.99–87.85] 0.03
	τ=0.90	24.81 [16.84–31.19] 0.08	3.93 [2.56–4.54] 0.17	3.75 [2.81–4.09] 0.23	10.88 [5.26–16.48] 0.11	52.22 [47.99–87.85] 0.03
	Final	0.00 [0.00–0.00] 0.00	0.00 [0.00–0.00] 0.00	0.00 [0.00–0.00] 0.00	0.00 [0.00–0.00] 0.00	0.00 [0.00–0.00] 0.00
DLO	Initial	307.44 [304.42–311.92] 1.00	23.25 [23.05–23.46] 1.00	15.72 [15.62–15.92] 1.00	101.36 [100.30–102.64] 1.00	1858.56 [1842.37–1883.50] 1.00
	τ=0.50	170.03 [168.52–172.78] 0.55	12.40 [12.17–12.61] 0.53	8.80 [8.70–9.00] 0.56	55.07 [54.25–56.71] 0.55	1028.04 [1005.09–1041.10] 0.55
	τ=0.75	84.97 [83.76–87.50] 0.28	6.38 [6.21–6.61] 0.27	4.63 [4.56–4.71] 0.30	28.20 [27.91–28.91] 0.28	518.43 [507.91–528.41] 0.28
	τ=0.90	34.33 [33.63–35.16] 0.11	2.55 [2.48–2.62] 0.11	1.92 [1.86–1.97] 0.12	11.34 [11.02–11.64] 0.11	207.99 [204.26–212.59] 0.11
	Final	0.00 [0.00–0.00] 0.00	0.00 [0.00–0.00] 0.00	0.00 [0.00–0.00] 0.00	0.00 [0.00–0.00] 0.00	0.00 [0.00–0.00] 0.00
PSO	Initial	308.89 [307.39–310.81] 1.00	23.17 [22.88–23.33] 1.00	15.98 [15.81–16.12] 1.00	101.39 [100.54–102.32] 1.00	1856.54 [1841.33–1867.11] 1.00
	τ=0.50	140.57 [138.15–144.76] 0.46	11.54 [11.04–11.74] 0.50	7.88 [7.53–8.19] 0.50	47.34 [45.89–49.08] 0.46	848.19 [842.86–865.10] 0.46
	τ=0.75	79.11 [67.19–81.52] 0.26	5.68 [5.36–5.86] 0.25	4.09 [3.82–4.20] 0.26	25.56 [21.26–26.93] 0.25	483.77 [391.29–504.66] 0.26
	τ=0.90	31.40 [29.96–32.34] 0.10	2.39 [2.19–2.46] 0.10	1.77 [1.75–1.80] 0.11	10.38 [9.86–10.65] 0.10	188.94 [178.20–196.32] 0.10
	Final	0.13 [0.10–0.18] 0.00	0.00 [0.00–0.00] 0.00	0.00 [0.00–0.00] 0.00	0.02 [0.01–0.03] 0.00	0.95 [0.67–1.22] 0.00

**Table 9:** Swarm Radius and Relative Contraction at Operational Diversity Loss Levels (50 Dimensions).

Metaheuristics	Threshold	Sphere	Rosenbrock	Rastrigin	Ackley	Griewank
RSA	Initial	399.67 [396.24–401.60] 1.00	29.98 [29.69–30.24] 1.00	20.45 [20.30–20.60] 1.00	131.56 [130.21–132.47] 1.00	2410.52 [2388.64–2430.40] 1.00
	τ=0.50	38.34 [24.30–60.10] 0.10	12.19 [11.03–13.84] 0.41	11.46 [10.88–12.24] 0.56	25.23 [20.06–34.02] 0.19	76.08 [64.62–100.90] 0.03
	τ=0.75	38.34 [24.30–60.10] 0.10	9.59 [6.72–12.08] 0.33	9.49 [9.03–10.15] 0.47	25.23 [20.06–34.02] 0.19	76.08 [64.62–100.90] 0.03
	τ=0.90	38.34 [24.30–60.10] 0.10	5.10 [3.89–6.65] 0.17	5.56 [4.56–6.19] 0.27	20.44 [15.84–24.16] 0.16	76.08 [64.62–100.90] 0.03
	Final	0.00 [0.00–0.00] 0.00	0.00 [0.00–0.00] 0.00	0.00 [0.00–0.00] 0.00	0.00 [0.00–0.00] 0.00	0.00 [0.00–0.00] 0.00
DLO	Initial	399.57 [398.29–404.72] 1.00	29.95 [29.85–30.20] 1.00	20.55 [20.39–20.71] 1.00	131.44 [130.52–132.22] 1.00	2394.37 [2382.71–2422.91] 1.00
	τ=0.50	223.30 [221.18–226.20] 0.56	16.19 [15.89–16.41] 0.54	11.60 [11.46–11.76] 0.56	73.30 [72.79–74.08] 0.56	1344.47 [1317.57–1367.31] 0.56
	τ=0.75	114.46 [111.13–116.66] 0.28	8.44 [8.27–8.58] 0.28	6.25 [6.08–6.40] 0.30	37.82 [37.26–38.58] 0.29	688.47 [677.82–695.67] 0.29
	τ=0.90	45.96 [44.69–47.05] 0.11	3.46 [3.35–3.50] 0.11	2.60 [2.46–2.71] 0.13	15.09 [14.75–15.40] 0.12	276.31 [267.76–281.55] 0.12
	Final	0.00 [0.00–0.00] 0.00	0.00 [0.00–0.00] 0.00	0.00 [0.00–0.00] 0.00	0.00 [0.00–0.00] 0.00	0.01 [0.00–0.02] 0.00
PSO	Initial	398.48 [395.84–403.97] 1.00	30.21 [29.85–30.40] 1.00	20.51 [20.33–20.61] 1.00	131.25 [130.47–132.50] 1.00	2408.33 [2386.52–2429.04] 1.00
	τ=0.50	185.54 [183.63–190.48] 0.47	14.91 [14.54–15.09] 0.49	10.29 [9.95–10.45] 0.50	63.43 [62.56–64.62] 0.48	1135.83 [1104.22–1155.18] 0.47
	τ=0.75	97.88 [83.83–108.36] 0.24	6.96 [6.81–7.20] 0.23	5.27 [5.00–5.51] 0.25	28.21 [27.55–28.94] 0.22	520.64 [491.85–650.51] 0.22
	τ=0.90	41.65 [39.86–43.71] 0.10	3.22 [2.79–3.47] 0.11	2.25 [2.19–2.29] 0.11	12.92 [12.51–13.63] 0.10	246.54 [239.06–256.52] 0.10
	Final	1.21 [0.87–1.42] 0.00	0.00 [0.00–0.00] 0.00	0.01 [0.01–0.02] 0.00	0.00 [0.00–0.00] 0.00	7.61 [6.23–9.03] 0.00

**Table 10:** Swarm Radius and Relative Contraction at Operational Diversity Loss Levels (100 Dimensions).

Metaheuristics	Threshold	Sphere	Rosenbrock	Rastrigin	Ackley	Griewank
RSA	Initial	567.41 [564.66–569.53] 1.00	42.54 [42.29–42.79] 1.00	28.95 [28.78–29.17] 1.00	185.84 [185.03–186.56] 1.00	3394.58 [3376.70–3417.67] 1.00
	τ=0.50	57.95 [37.75–85.31] 0.10	17.68 [16.01–18.55] 0.42	15.86 [15.10–16.99] 0.54	41.21 [30.68–47.79] 0.22	127.55 [102.09–177.13] 0.04
	τ=0.75	57.95 [37.75–85.31] 0.10	13.90 [8.85–16.04] 0.33	13.49 [12.59–14.88] 0.47	41.21 [30.68–47.79] 0.22	127.55 [102.09–177.13] 0.04
	τ=0.90	57.95 [37.75–85.31] 0.10	7.70 [6.82–8.99] 0.18	8.57 [7.87–9.20] 0.30	24.93 [13.16–30.68] 0.13	127.55 [102.09–177.13] 0.04
	Final	0.00 [0.00–0.00] 0.00	0.00 [0.00–0.00] 0.00	0.00 [0.00–0.00] 0.00	0.00 [0.00–0.00] 0.00	0.00 [0.00–0.00] 0.00
DLO	Initial	568.14 [564.08–570.47] 1.00	42.50 [42.20–42.89] 1.00	29.10 [28.93–29.25] 1.00	186.25 [185.47–186.91] 1.00	3403.59 [3381.85–3425.17] 1.00
	τ=0.50	320.00 [316.94–322.05] 0.56	23.40 [23.12–23.51] 0.55	16.59 [16.46–16.76] 0.57	105.12 [104.50–105.61] 0.57	1907.99 [1881.18–1936.62] 0.56
	τ=0.75	170.35 [167.79–173.04] 0.30	12.30 [12.12–12.47] 0.29	9.07 [8.92–9.24] 0.31	55.62 [54.98–56.76] 0.30	1023.68 [1004.35–1034.37] 0.30
	τ=0.90	69.66 [68.14–71.14] 0.12	5.03 [4.98–5.13] 0.12	4.16 [3.97–4.31] 0.14	22.77 [22.05–23.17] 0.12	416.20 [399.33–419.34] 0.12
	Final	0.00 [0.00–0.01] 0.00	0.00 [0.00–0.00] 0.00	0.00 [0.00–0.00] 0.00	0.00 [0.00–0.00] 0.00	0.02 [0.01–0.03] 0.00
PSO	Initial	567.01 [563.99–568.67] 1.00	42.48 [42.36–42.93] 1.00	29.02 [28.95–29.19] 1.00	185.68 [184.91–187.42] 1.00	3402.45 [3384.01–3416.92] 1.00
	τ=0.50	269.30 [267.18–273.42] 0.48	21.22 [20.79–21.58] 0.50	14.52 [14.31–14.74] 0.50	90.98 [89.61–92.22] 0.49	1628.59 [1611.35–1643.87] 0.48
	τ=0.75	120.53 [118.40–124.39] 0.21	9.92 [9.72–10.29] 0.23	7.73 [7.13–7.90] 0.26	41.76 [40.51–43.03] 0.22	726.69 [715.08–742.92] 0.21
	τ=0.90	62.90 [58.41–64.28] 0.11	4.72 [4.17–4.87] 0.11	3.26 [3.17–3.33] 0.11	20.12 [19.24–20.85] 0.11	347.56 [343.25–355.82] 0.10
	Final	5.36 [4.95–5.87] 0.01	0.00 [0.00–0.01] 0.00	0.09 [0.08–0.11] 0.00	0.00 [0.00–0.00] 0.00	33.53 [27.84–37.68] 0.01

**Table 11:** Fitness Improvement Fraction at Operational Diversity Loss Levels (30 Dimensions).

Metaheuristics	Threshold	Sphere	Rosenbrock	Rastrigin	Ackley	Griewank
RSA	Initial	66,926.98 [61,428.89–70,167.79] 0.00	1,544,066.00 [1,353,948.00–1,805,281.00] 0.00	452.02 [420.84–467.40] 0.00	20.77 [20.67–20.89] 0.00	610.12 [561.14–630.34] 0.00
	τ=0.50	64,569.12 [59,620.14–67,487.59] 0.04	1,308,303.00 [1,151,554.00–1,509,715.00] 0.13	445.17 [420.59–459.83] 0.00	20.69 [20.49–20.77] 0.00	587.07 [545.11–603.37] 0.04
	75% Loss	64,569.12 [59,620.14–67,487.59] 0.04	1,178,626.50 [992,269.80–1,386,314.00] 0.23	428.12 [407.70–444.04] 0.02	20.69 [20.49–20.77] 0.00	587.07 [545.11–603.37] 0.04
	τ=0.90	64,569.12 [59,620.14–67,487.59] 0.04	1,017,104.00 [929,239.30–1,211,995.00] 0.32	422.19 [400.10–442.79] 0.05	20.68 [20.46–20.77] 0.01	587.07 [545.11–603.37] 0.04
	Final	0.00 [0.00–0.00] 1.00	29.00 [29.00–29.00] 1.00	0.00 [0.00–0.00] 1.00	0.00 [0.00–0.00] 1.00	0.00 [0.00–0.00] 1.00
DLO	Initial	69,568.10 [62,238.00–74,272.66] 0.00	1,713,820.50 [1,484,453.00–1,964,631.00] 0.00	444.91 [423.40–461.18] 0.00	20.78 [20.66–20.90] 0.00	621.22 [594.46–659.29] 0.00
	τ=0.50	15,818.92 [13,727.85–19,014.26] 0.75	82,993.78 [58,963.41–95,265.97] 0.96	273.98 [255.58–292.24] 0.39	17.35 [16.92–17.98] 0.16	155.88 [138.00–172.55] 0.75
	τ=0.75	3915.26 [3370.61–4609.46] 0.94	6002.01 [4755.86–7211.70] 1.00	169.89 [158.90–189.28] 0.63	12.41 [11.34–13.21] 0.41	42.25 [36.35–46.94] 0.93
	τ=0.90	684.26 [474.79–862.18] 0.99	502.95 [425.65–543.57] 1.00	81.26 [26.45–118.40] 0.82	6.93 [6.26–7.62] 0.67	6.95 [6.23–7.48] 0.99
	Final	0.00 [0.00–0.00] 1.00	27.32 [26.94–27.65] 1.00	0.01 [0.00–0.04] 1.00	0.00 [0.00–0.00] 1.00	0.00 [0.00–0.00] 1.00
PSO	Initial	66,808.77 [59,702.12–73,860.86] 0.00	1,464,229.50 [1,088,678.00–1,898,686.00] 0.00	449.48 [432.36–469.31] 0.00	20.76 [20.64–20.86] 0.00	616.22 [585.01–644.56] 0.00
	τ=0.50	18,943.94 [17,615.66–20,483.81] 0.71	320,923.15 [293,164.80–345,705.10] 0.78	296.93 [282.07–306.40] 0.36	17.86 [17.54–18.08] 0.15	175.20 [165.49–185.36] 0.72
	τ=0.75	7686.63 [5863.07–9328.92] 0.88	85,127.46 [70,590.20–110,734.20] 0.94	247.12 [236.27–262.05] 0.48	14.67 [13.46–15.02] 0.31	72.33 [64.26–80.74] 0.88
	τ=0.90	2938.66 [2360.95–3539.90] 0.95	38,499.57 [29,880.17–48,071.64] 0.97	192.98 [184.93–210.45] 0.62	11.16 [10.68–11.88] 0.48	29.77 [25.43–34.24] 0.95
	Final	0.04 [0.02–0.06] 1.00	282.19 [227.14–366.59] 1.00	32.84 [26.86–44.77] 1.00	1.42 [0.05–1.90] 1.00	0.05 [0.03–0.07] 1.00

**Table 12:** Fitness Improvement Fraction at Operational Diversity Loss Levels (50 Dimensions).

Metaheuristics	Threshold	Sphere	Rosenbrock	Rastrigin	Ackley	Griewank
RSA	Initial	123,652.30 [113,485.30–131,058.50] 0.00	3,474,275.00 [3,051,933.00–3,652,954.00] 0.00	773.67 [755.30–809.34] 0.00	20.91 [20.83–20.97] 0.00	1145.09 [1064.27–1197.88] 0.00
	τ=0.50	120,010.90 [109,530.40–126,822.50] 0.03	3,050,183.00 [2,679,476.00–3,319,852.00] 0.10	766.86 [740.89–808.18] 0.00	20.79 [20.72–20.87] 0.00	1105.94 [1022.16–1160.63] 0.04
	τ=0.75	120,010.90 [109,530.40–126,822.50] 0.03	2,657,432.50 [2,493,615.00–2,890,004.00] 0.22	755.82 [730.96–778.25] 0.03	20.79 [20.72–20.87] 0.00	1105.94 [1022.16–1160.63] 0.04
	τ=0.90	120,010.90 [109,530.40–126,822.50] 0.03	2,356,761.00 [1,919,696.00–2,551,960.00] 0.31	753.32 [713.73–763.47] 0.05	20.79 [20.72–20.87] 0.00	1105.94 [1022.16–1160.63] 0.04
	Final	0.00 [0.00–0.00] 1.00	49.00 [49.00–49.00] 1.00	0.00 [0.00–0.00] 1.00	0.00 [0.00–0.00] 1.00	0.00 [0.00–0.00] 1.00
DLO	Initial	125,894.80 [115,968.00–132,111.40] 0.00	3,511,031.50 [3,030,165.00–3,756,559.00] 0.00	790.32 [766.32–816.85] 0.00	20.89 [20.85–21.00] 0.00	1138.71 [1052.26–1187.55] 0.00
	τ=0.50	31,386.86 [20,712.15–37,210.07] 0.74	233,441.15 [126,577.20–260,528.40] 0.93	499.00 [478.68–520.06] 0.37	17.68 [17.03–18.64] 0.15	307.22 [248.43–329.71] 0.73
	τ=0.75	7969.94 [6083.36–10,039.38] 0.93	14,961.04 [11,117.96–18,321.84] 1.00	352.78 [308.86–380.69] 0.57	13.20 [12.02–14.06] 0.37	76.97 [71.67–80.90] 0.93
	τ=0.90	1401.47 [904.42–1824.14] 0.99	1000.55 [815.09–1153.10] 1.00	172.81 [137.57–236.54] 0.79	7.60 [6.72–8.38] 0.64	13.48 [12.10–14.45] 0.99
	Final	0.00 [0.00–0.00] 1.00	48.06 [47.76–48.24] 1.00	0.00 [0.00–0.01] 1.00	0.00 [0.00–0.00] 1.00	0.00 [0.00–0.00] 1.00
PSO	Initial	128,674.25 [116,373.60–134,020.60] 0.00	3,600,185.00 [2,973,449.00–3,919,156.00] 0.00	784.63 [763.27–800.14] 0.00	20.92 [20.85–20.98] 0.00	1151.63 [1101.58–1210.52] 0.00
	τ=0.50	36,193.78 [34,096.26–37,521.62] 0.71	715,935.35 [663,763.80–767,712.80] 0.81	532.65 [520.59–554.50] 0.34	18.40 [18.21–18.58] 0.20	323.70 [308.68–340.02] 0.72
	τ=0.75	13,505.03 [11,862.26–16,427.41] 0.89	210,250.45 [173,649.00–233,335.70] 0.94	448.21 [434.81–459.66] 0.46	14.79 [14.37–15.17] 0.48	126.88 [100.11–133.74] 0.89
	τ=0.90	6814.65 [5824.72–7655.31] 0.95	113,893.15 [85,630.30–136,259.90] 0.97	370.65 [342.05–399.49] 0.57	12.68 [12.27–13.02] 0.65	62.54 [54.14–71.31] 0.95
	Final	8.18 [4.94–11.88] 1.00	3953.77 [2602.57–5751.44] 1.00	56.89 [47.84–66.73] 1.00	8.28 [7.45–8.76] 1.00	1.08 [1.03–1.13] 1.00

**Table 13:** Fitness Improvement Fraction at Operational Diversity Loss Levels (100 Dimensions).

Metaheuristics	Threshold	Sphere	Rosenbrock	Rastrigin	Ackley	Griewank
RSA	Initial	270,800.65 [263,924.00–279,045.60] 0.00	8,779,376.50 [7,972,207.00–9,489,687.00] 0.00	1641.46 [1615.70–1653.33] 0.00	21.02 [20.96–21.07] 0.00	2491.99 [2333.73–2564.06] 0.00
	τ=0.50	263,233.10 [256,741.60–272,269.40] 0.03	7,861,037.50 [7,386,787.00–8,273,911.00] 0.08	1626.61 [1604.25–1653.33] 0.00	20.97 [20.92–21.02] 0.00	2409.37 [2263.09–2493.32] 0.03
	τ=0.75	263,233.10 [256,741.60–272,269.40] 0.03	7,033,748.50 [6,247,371.00–7,678,253.00] 0.20	1626.61 [1604.25–1650.08] 0.00	20.97 [20.92–21.02] 0.00	2409.37 [2263.09–2493.32] 0.03
	τ=0.90	263,233.10 [256,741.60–272,269.40] 0.03	6,422,694.50 [5,564,657.00–6,836,570.00] 0.27	1612.38 [1593.90–1650.08] 0.00	20.96 [20.92–21.02] 0.00	2409.37 [2263.09–2493.32] 0.03
	Final	0.00 [0.00–0.00] 1.00	99.00 [99.00–99.00] 1.00	0.00 [0.00–0.00] 1.00	0.00 [0.00–0.00] 1.00	0.00 [0.00–0.00] 1.00
DLO	Initial	278,161.15 [267,484.20–283,315.20] 0.00	9,010,502.50 [7,831,928.00–9,451,481.00] 0.00	1669.26 [1631.31–1693.29] 0.00	21.03 [20.99–21.05] 0.00	2443.78 [2325.20–2554.91] 0.00
	τ=0.50	68,655.64 [49,375.97–86,505.09] 0.76	463,115.15 [354,783.10–546,360.50] 0.95	1087.22 [1027.57–1109.20] 0.36	18.21 [17.59–18.83] 0.13	614.49 [547.87–654.44] 0.76
	τ=0.75	19,945.97 [15,115.61–25,690.49] 0.92	45,812.74 [39,391.81–58,775.20] 0.99	779.67 [744.33–850.33] 0.53	13.94 [12.56–14.89] 0.34	180.20 [165.41–209.68] 0.93
	τ=0.90	3434.92 [2684.04–4090.48] 0.99	3122.74 [2581.67–3714.79] 1.00	433.09 [337.76–498.62] 0.73	7.96 [7.07–8.78] 0.62	29.03 [26.62–32.17] 0.99
	Final	0.03 [0.02–0.03] 1.00	98.20 [98.10–98.39] 1.00	0.00 [0.00–0.01] 1.00	0.00 [0.00–0.01] 1.00	0.02 [0.01–0.02] 1.00
PSO	Initial	271,262.60 [261,815.30–281,774.60] 0.00	8,499,876.00 [7,877,410.00–9,247,292.00] 0.00	1656.95 [1635.58–1696.66] 0.00	21.02 [20.98–21.08] 0.00	2473.36 [2396.29–2566.98] 0.00
	τ=0.50	79,280.71 [75,350.17–81,540.37] 0.71	1,785,727.50 [1,634,050.00–1,905,484.00] 0.80	1143.07 [1114.88–1158.21] 0.34	18.60 [18.55–18.73] 0.23	713.35 [699.75–735.17] 0.71
	τ=0.75	26,860.84 [24,313.36–28,340.01] 0.90	583,312.30 [527,026.70–622,645.70] 0.94	981.42 [937.12–1003.08] 0.45	15.15 [14.92–15.47] 0.55	244.45 [224.51–258.42] 0.90
	τ=0.90	17,830.77 [15,669.53–19,942.52] 0.94	346,234.70 [319,865.80–384,458.70] 0.96	864.04 [827.06–881.70] 0.54	13.50 [13.18–13.97] 0.72	161.71 [146.20–177.32] 0.94
	Final	555.72 [450.33–741.61] 1.00	61,534.62 [48,422.67–68,179.61] 1.00	157.79 [140.10–181.87] 1.00	10.56 [9.97–10.97] 1.00	5.64 [4.44–6.60] 1.00

## Data Availability

The original contributions presented in the study are included in the article; further inquiries can be directed to the corresponding authors.
